# Attenuating Adaptive VEGF-A and IL8 Signaling Restores Durable Tumor Control in AR Antagonist–Treated Prostate Cancers

**DOI:** 10.1158/1541-7786.MCR-21-0780

**Published:** 2022-03-18

**Authors:** Pamela J. Maxwell, Melanie McKechnie, Christopher W. Armstrong, Judith M. Manley, Chee Wee Ong, Jenny Worthington, Ian G. Mills, Daniel B. Longley, James P. Quigley, Amina Zoubeidi, Johann S. de Bono, Elena Deryugina, Melissa J. LaBonte, David J.J. Waugh

**Affiliations:** 1 Movember FASTMAN Centre of Excellence, Patrick G Johnston Centre for Cancer Research, School of Medicine, Dentistry and Biomedical Sciences, Queen's University Belfast, Belfast, Northern Ireland, United Kingdom.; 2 Axis Biosciences, Coleraine, Northern Ireland, United Kingdom.; 3 Department of Cell Biology, The Scripps Research Institute, La Jolla, California.; 4 The Vancouver Prostate Centre, University of British Columbia, Vancouver, Canada.; 5 Division of Clinical Studies, The Institute of Cancer Research and The Royal Marsden NHS Foundation Trust, Sutton, United Kingdom.; 6 School of Biomedical Sciences, Queensland University of Technology, Brisbane Australia.; 7 Translational Research Institute, Princess Alexandra Hospital, Brisbane, Australia.

## Abstract

Inhibiting androgen signaling using androgen signaling inhibitors (ASI) remains the primary treatment for castrate-resistant prostate cancer. Acquired resistance to androgen receptor (AR)-targeted therapy represents a major impediment to durable clinical response. Understanding resistance mechanisms, including the role of AR expressed in other cell types within the tumor microenvironment, will extend the clinical benefit of AR-targeted therapy. Here, we show the ASI enzalutamide induces vascular catastrophe and promotes hypoxia and microenvironment adaptation. We characterize treatment-induced hypoxia, and subsequent induction of angiogenesis, as novel mechanisms of relapse to enzalutamide, highlighting the importance of two hypoxia-regulated cytokines in underpinning relapse. We confirmed AR expression in CD34^+^ vascular endothelium of biopsy tissue and human vascular endothelial cells (HVEC). Enzalutamide attenuated angiogenic tubule formation and induced cytotoxicity in HVECs *in vitro*, and rapidly induced sustained hypoxia in LNCaP xenografts. Subsequent reoxygenation, following prolonged enzalutamide treatment, was associated with increased tumor vessel density and accelerated tumor growth. Hypoxia increased AR expression and transcriptional activity in prostate cells *in vitro*. Coinhibition of IL8 and VEGF-A restored tumor response in the presence of enzalutamide, confirming the functional importance of their elevated expression in enzalutamide-resistant models. Moreover, coinhibition of IL8 and VEGF-A resulted in a durable, effective resolution of enzalutamide-sensitive prostate tumors. We conclude that concurrent inhibition of two hypoxia-induced factors, IL8 and VEGF-A, prolongs tumor sensitivity to enzalutamide in preclinical models and may delay the onset of enzalutamide resistance.

**Implications:**

Targeting hypoxia-induced signaling may extend the therapeutic benefit of enzalutamide, providing an improved treatment strategy for patients with resistant disease.

## Introduction

The inhibition of androgen signaling, through use of androgen ablating agents and androgen signaling inhibitors (ASI), remains the therapeutic mainstay in the treatment of primary and metastatic prostate cancer, respectively (1). The ASI enzalutamide blocks androgen binding, nuclear translocation, and androgen-driven gene expression (1). The ARCHES, ENZAMET, and AFFIRM trials have demonstrated a survival benefit for enzalutamide in both the metastatic castrate-sensitive and castrate-resistant prostate cancer (CRPC), underpinning enzalutamide's widespread clinical use (2–4). Despite initial patient response, enzalutamide resistance (EnzR) is a major occurrence and has been attributed to the retention of active androgen receptor (AR) signaling in androgen-depleted conditions, via amplification/overexpression of AR, elevated intra-prostatic androgen synthesis, or expression of AR splice variants (5). However, the importance of the tumor microenvironment (TME) and the full impacts of perturbing androgen signaling upon other tumor-resident cell types has rarely been considered in the context of EnzR. Expression of the AR is not exclusive to the epithelial cells of the prostate gland and is expressed in other compartments of the prostate stroma, including cancer-associated fibroblasts (CAF). Indeed, androgen signaling is a key contributor to glandular development of the prostate gland and carcinogenesis.

Impacts of modulating androgen signaling using ASIs upon the prostate TME have been reported, including observation of rapidly decreasing prostatic blood flow and reduction in microvessel density (MVD) *in vivo* (6). Rapid vascular atrophy and reduced MVD have been observed in engrafted human prostate tumors following androgen withdrawal caused, in part, by human vascular endothelial cell (HVEC) apoptosis (7). Similarly, the prior generation AR inhibitor Bicalutamide induced a rapid and sustained intratumoral hypoxia, followed by angiogenesis *in vivo* (8). Transcriptomic profiling of these bicalutamide-treated tumors identified adaptive hypoxia-associated responses *in vivo*, including elevated gene expression of the pro-angiogenic factors IL8 and VEGF-A (VEGF) *in vivo* (8). We have adopted the hypothesis that ASI treatment-associated hypoxia and the ensuing paracrine and autocrine signaling of IL8 and VEGF defines a further mechanism of relapse to ASI's and accelerates the outgrowth of resistant disease. This study aimed to characterize the dynamic response of prostate tumors to enzalutamide, a current generation AR antagonist, and to demonstrate the functional significance of IL8 and VEGF signaling in modulating the adaptive tumor response.

## Materials and Methods

### Cell culture

PC3 (PPID:CVCL_0035; ATCC CRL-1435) and LNCaP (RRID:CVCL_1379; ATCC CRL-1740) were cultured in standard culture media as recommended by ATCC (9). C4-2B (RRID:CVCL_4784) and CWR-R1 (RRID:CVCL_4833) were maintained in RPMI1640 with 10% fetal calf serum. Enzalutamide-sensitive (LNCaP-Par, CWR-R1-Par) and -resistant (LNCaP-EnzR, CWR-R1-EnzR) cells (obtained from Prof. Vander Griend, University of Chicago, Chicago, Illinois) were cultured as described (10). Human umbilival vein endothelial cells (HUVEC; RRID:CVCL_2959; ATCC CRL-1730) cells were maintained in Endothelial Cell Growth Medium (#CC-3162; Lonza). Hypoxic cells were cultured as described (11). Human IL8 monoclonal antibody (RRID:AB_2249110; #MAB208), human VEGF mAb (RRID:AB_354341; #AB293NA) and isotype-matched IgG (RRID:AB_357344; #MAB002) were obtained from R&D Systems. MDV3100 (#S1250) was obtained from Selleckchem. Cells were authenticated by short tandem repeat analysis and were regularly tested for *Mycoplasma* by MycAlert (Lonza) according to the manufacturer's instructions.

### Cell viability

Cell viability was assessed by MTT assay as described (11). Briefly, cells were seeded in 96-well plates, allowed to adhere and then treated for 72 hours. MTT (5 mg/mL, Sigma) was added and cells were further incubated for 3 hours. Culture media was removed and the formazan crystals were reabsorbed in DMSO. Cell viability was determined by reading the absorbance of each well at 570 nm.

### qRT-PCR

Samples were prepared and analyzed as described (11). Briefly, RNA was harvested using TriPure (11667157001; Roche) and cDNA synthesized using 2-μg RNA was reverse transcribing using MMLV-RT (#28025013). qRT-PCR reactions were performed on the Lightcycler480 instrument using SYBR Green Master (Roche) with standard cycling procedures and relative quantification of mRNA levels was calculated relative to the standard curve and 18S housekeeping gene. Primer sequences for *18S*, *BCL2*, *CAIX*, *CXCL8*, *VEGFA* have been reported (11). Other primer sequences are available in Supplementary Methods.

### Immunoblotting

Immunoblotting was as described (12). Briefly, cells were seeded, treated as required and protein was harvested using RIPA buffer and a cocktail of protease/phosphates inhibitors (#A32963, Thermo Scientific). Protein was quantified using the BCA assay (#23227, Thermo Fisher Scientific) and 20-μg protein was resolved on appropriate SDS-polyacrylamide gels depending on the size of the protein of interest. Proteins were transferred onto Hybond-P (#GE10600023, Sigma) and probed using appropriate antibodies (see Supplementary Methods). Immunoreactivity was detected using the SuperSignal West Pico Detection Kit (#34577, Thermo Fisher Scientific). The level of protein signal was quantified using Image J.

### ELISA

Secreted IL8 (#M1918; Pelikine, Mast Group) and VEGF-A (#DY293B; R&D Systems) were detected by ELISA according to the manufacturer's instructions. Briefly, cells were plated and treated as indicated and the media was collected and the cell number was recorded using a Coulter Counter (Beckman Coulter). Standards and samples were added to a 96-well plate that had been coated with the appropriate capture antibody and incubated as required. Colorimetric detection was achieved using streptavidin-HRP and absorbance at 450 nm (reference 570 nm). A standard curve was generated and the quantity of IL8 or VEGFA in each sample was calculated and correlated to cell number.

### Immunofluorescence

Cells were seeded on coverslips, fixed, permeabilized, blocked, and visualized (anti-AR; RRID:AB_310214; #PG-21, Millipore) and Alexa Fluor 568 (RRID:AB_10563566; #A11036; Molecular Probes) antibodies. Nuclei were visualized using ProLong Gold Antifade with DAPI (#P36941; Thermo Scientific). Cells were viewed under a Nikon Eclipse Ti-S fluorescent microscope and images captured using NIS-Elements software.

### RNA sequencing

RNA sequencing analysis was performed using established protocols as described (13). Array data are available at Gene Expression Omnibus accession number GSE55345.

### IHC

IHC was performed for AR as described (14). Briefly, cells were seeded on coverslips, cultured, and treated as indicated. Following treatment cells were fixed, permeabilized, and blocked overnight. AR was visualized using primary anti-AR antibody (RRID:AB_307266; #ab9474; Abcam) and CD31 (RRID:AB_726362; #ab28364; Abcam). ProLong Gold Antifade Reagent with DAPI (#P36941, Thermo Fisher Scientific) was used to visualize the nuclei. Cells were viewed under a Nikon Eclipse Ti-S fluorescent microscope and images captured using NIS-Elements software.

### *In vitro* angiogenesis assay

V2A angiogenesis assay (#ZHA-4000; Cellworks) was carried out as described (15). Briefly, cells were allowed to settle for 4 days prior to treatment. Treatments were replenished every 2 days for 10 days. For treatments involving conditioned media (CM), prostate cancer cells were treated as indicated for 24 hours and then the media was harvested and stored at –20°C. Vessels were visualized at 4× magnification using an Olympus CKX41 light microscope. Vessel density and number of junctions was measured by AngioSys 2.0 software (Cellworks).

### Chick embryo experimental metastatic assay

Experimental metastasis assays were performed in chick embryos as described (16). PC3 cells were injected directly into the chorioallantoic vein of a day 10 chick embryo (1×10^5^cells/embryo). 10-μmol/L enzalutamide was delivered intravenously on days 1 and 2 post-tumor cell inoculation. On day 5, the lower cam was excised and analyzed with human-specific *Alu-*qPCR as described (16) for number of human cells.

### Real-time *Alu* PCR for quantitative detection of human tumor cells

Human cells within the chick embryo tissue were detected by real-time *Alu* PCR, as described (17). Briefly, genomic DNA was extracted from harvested tissue. Human *Alu* sequences were amplified by real-time PCR using 30-ng genomic DNA in a 10-μL reaction containing 2-mmol/L MgCl_2_, 200-μmol/L deoxynucleotide triphosphate, 0.4-unit Platinum *Taq* polymerase (Invitrogen) 1:10^5^ SYBR Green dye (Molecular Probes, Eugene, Oregon) and 0.4 μmol/L of each *Alu* primers: ense (5′-ACGCCTGTAATCCCAGCACTT-3′) and *Alu* antisense (5′-TCGCCCAGGCTGGGTGCA-3′). The actual number of tumor cells present in the chorioallantoic membrane (CAM) was determined using a standard curve generated by serial dilution of human tumor cells as previously described (17).

### *In vivo* models

*In vivo* experiments were conducted per the Animal (Scientific Procedures) Act 1986 and the United Kingdom Co-ordinating Committee on Cancer Research (UKCCCR) Guidelines (2010) for the welfare of animals in experimental models. Drug administration and study protocols were as described in Supplementary Methods.

### Statistical analysis

Statistical significance was determined using a two-tailed Student *t* test, Mann–Whitney U test or 2-way ANOVA with Bonferroni post-tests, as appropriate using GraphPad Prism software. All experiments were done three times and the total number of experiments and samples are indicated in the appropriate figure legends. Data are presented as the mean ± SEM.

### Ethics approval and consent to participate

All animal experiments complied with the Animal (Scientific Procedures) Act 1986 and the UKCCCR Guidelines (2010) for the welfare of animals in experimental models and were approved by the Queen's University Belfast's Animal Welfare Ethical Review Body.

### Availability of data and materials

The datasets used and/or analyzed during the current study are available from the corresponding author on reasonable request.

## Results

### Enzalutamide promotes hypoxia and delays angiogenesis through endothelial cell vascular catastrophe

**Figure 1.**
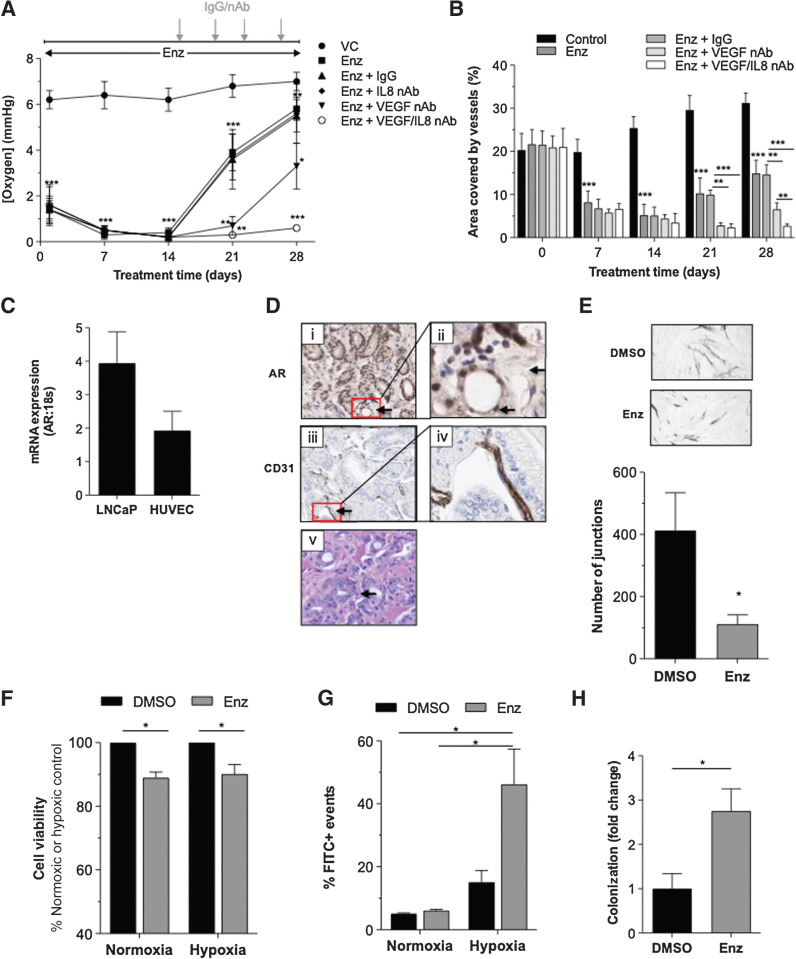
*Enzalutamide treatment directly modulates tumor vasculature.* LNCaP *in vivo* tumors (*N* = 4/group) treated with enzalutamide (Enz) in the presence or absence of anti-IL8 nAb (50 μg/mL) and/or anti-VEGF nAb (100 μg/mL) for 28 days and measured time-dependent changes in (**A**) intratumoral oxygenation concentration (mmHg) and (**B**) tumor vessel density. Values shown are mean±SD. Treatment schematic is illustrated above the graph. Stereological methods were used to analyze the change in vessel density over time in all treatment groups and values were used to calculate percentage area covered by vessels. **C,** qRT-PCR data demonstrating detectable basal AR expression in LNCaP and HUVEC cells. Data shown are mean±SEM; *N* = 4 experiments. **D,** Representative images of prostate tumor stained for (i, ii) AR (20X and 40X), (iii, iv) CD31 (20X and 40X), and (v) hematoxylin and eosin (20X). Endothelial cells (EC) and vessels are marked by black arrows in the prostate tumor. **E,** Effect of 10 μmol/L Enz on *in vitro* tubule formation over 10 days. The number of junctions was measured using AngioSys 2.0 software. Data presented are mean±SEM of *N* = 8 fields of view; *N* = 4 experiments (**F**) Effect of 10 μmol/L Enz on viability of HUVEC cells following either 72-hour normoxic or hypoxic conditions. Data shown are mean±SEM; *N* = 3 experiments. **G,** Effect of 10 μmol/L Enz on apoptosis in HUVEC cells following either 72-hour normoxic or hypoxic conditions. Data shown are mean±SEM; *N* = 3 experiments. **H,** Effect of 10 μmol/L Enz on the colonization of PC3 cells over 5 days in the *in ovo* assay. Data shown are mean ± SEM of *N* = 17 embryos for DMSO and *N* = 23 embryos for the Enz. For all data, control cells were treated with equivalent volume of DMSO and statistically significant differences were determined using a Student two-tailed *t* test or Mann–Whitney U test: *, *P <* 0.05; **, *P <* 0.01; ***, *P <* 0.001.

Experiments were conducted to characterize the effect of enzalutamide (MDV3100) on tumor oxygenation and MVD. Using LNCaP xenografts, we observed that enzalutamide administration induced a rapid drop in tumor oxygenation reaching a nadir at 14 days, after which oxygen levels were shown to gradually increase towards pretreatment levels (Fig. 1A). To determine the impacts of inhibiting either hypoxia-induced IL8 and/or VEGF signaling, neutralizing antibodies (nAb) to these cytokines were administered following 14 days of enzalutamide therapy. Although administration of the anti-IL8 nAb alone had no effect on enzalutamide-induced oxygen dynamics within the tumor, the anti-VEGF nAb retarded, but did not prevent, the restoration of oxygen tension in these tumors. Importantly, coadministration of anti-VEGF/anti-IL8 nAb's with enzalutamide from treatment day 14 resulted in a sustained attenuation of tumor oxygenation for the duration of the study (*P* < 0.001; Fig. 1A).

The tumor area covered by vessels was assessed as part of the study. Coverage was 21% ± 3% at treatment initiation and increased by 10% in vehicle-treated tumors by day 28 (Fig. 1B). Consistent with disruption of the vascular structure, treatment with enzalutamide significantly reduced vessel coverage between days 7 (8.2% ± 2.3%, *P* = 0.001) and 14 (5.2% ± 2.2%; *P* < 0.0001; statistical analysis shown in Supplementary Table S1). This trend was reversed at days 21 and 28 (10.3% ± 3.1%, day 21; 14.7% ± 2.6%, day 28), where increasing vessel density is supportive of an angiogenic response and consistent with prior oxygen tension recordings (Fig. 1A). In contrast, MVD remained suppressed at day 28 in the combined anti-VEGF+enzalutamide cohort (6.6% ± 1.3%; *P* < 0.0001 vs. IgG/enzalutamide; Supplementary Table S1) and was further suppressed in response to the anti-VEGF/anti-IL8+enzalutamide treatment (2.7% ± 0.3%; *P =* 0.009 vs. anti-VEGF+enzalutamide).

We next sought to confirm AR expression in the vascular endothelium. Consistent with prior reports (18), we detected AR mRNA in the human vascular endothelial cells (HUVEC; Fig. 1C) and AR expression in HVECs of human prostate tissue (Fig. 1D). Analysis of publicly available data also confirmed AR gene expression in HUVEC and HMEC cells (GSE26312; Supplementary Fig. S1). Furthermore, consistent with the observed vascular catastrophe *in vivo* (Fig. 1A), *in vitro* enzalutamide treatment in the HVEC tubule formation assay (Fig. 1E), reduced HUVEC viability by 10% under both normoxic and hypoxic conditions (*P* < 0.05; Fig. 1F), with increased detection of apoptosis under hypoxia (Fig. 1G).

The effect of enzalutamide on tumor vasculature was further investigated *in vivo* using the chicken CAM model, in which distal tumor colonization depends on the integrity of the endothelial barrier at the site of extravasation. We specifically employed AR-null, enzalutamide-insensitive PC3 cells (Supplementary Fig. S2A) in these experiments to place emphasis on the effects of enzalutamide on the endothelium as opposed to tumor cells. As an initial control experiment, CM harvested from enzalutamide-treated PC3 cells had no destructive effect on tubule formation *in vitro* suggesting the absence of tumor-derived factors that negatively affected HVEC viability (Supplementary Fig. S2B). However, enzalutamide administration to the CAM model significantly enhanced PC3 metastatic colonization in the model (*P =* 0.011; Fig. 1H). This result is consistent with enzalutamide affecting vascular integrity, including the endothelial barrier at sites of extravasation.

### Hypoxia potentiates AR pathway expression and activity in prostate cancer cells

**Figure 2.**
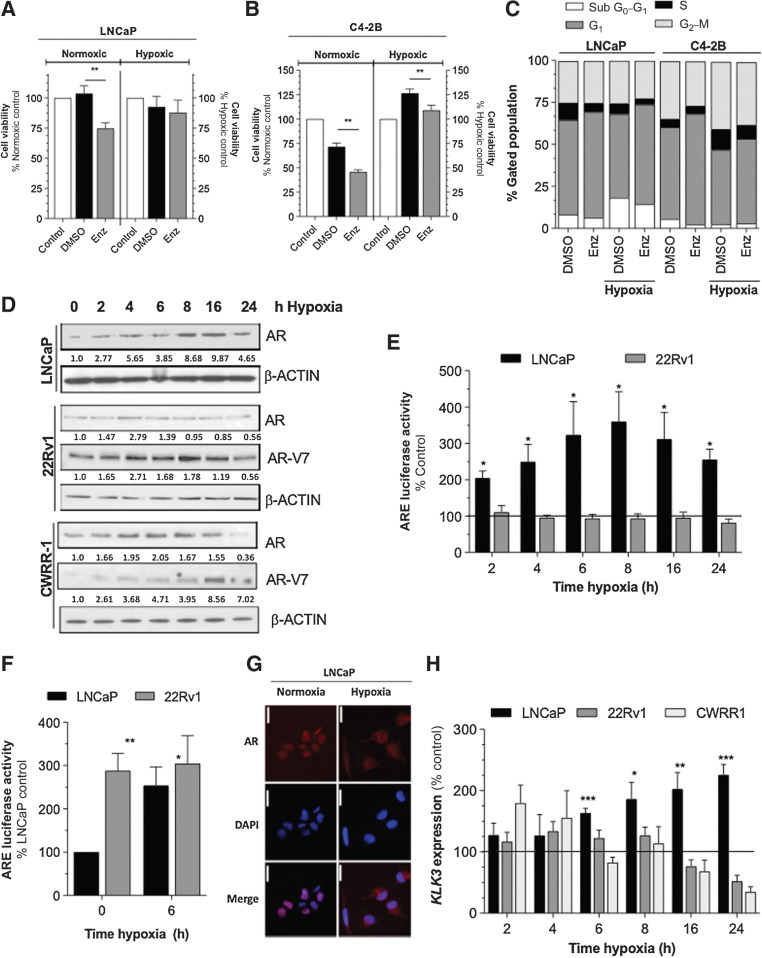
Treatment-mediated hypoxia-promoted VEGF and IL8 signaling influences AR expression and activity, angiogenesis and enzalutamide response. **A** and **B,** Effect of 10 μmol/L enzalutamide (Enz) on viability of LNCaP (**A**) or C4-2B (**B**) cells under normoxia and hypoxia for 72 hours. Control cells are presented as 100% for either untreated normoxic cells (left y-axis) or cells treated with hypoxia alone (right y-axis). As a control, were treated with an equivalent volume of DMSO. Data shown are mean ± SEM of *N* = 3 experiments. **C,** Cell-cycle analysis of 10 μmol/L Enz treated LNCaP and C4–2B cells following 72 hours. Control cells were treated with an equivalent volume of DMSO. Data are mean±SEM of *N* = 4 experiments. **D,** Effect of hypoxia on expression of the AR in LNCaP cells (*top*), and AR_FL_ and AR-V7 expression in 22Rv1 (*middle*), and CWR-R1 (*bottom*) cells. Blots shown are representative of *N* = 3 experiments. Equal loading was assessed using β-Actin. Relative expression was determined by densitometry using Image J software. **E,** Luciferase reporter assay demonstrating the effect of 2 to 24-hour hypoxia on AR transcriptional activity in LNCaP and 22Rv1 cells. Data are mean±SEM of *N* = 3 experiments. **F,** Luciferase reporter assay demonstrating the effect of hypoxia (6 hours) on AR transcriptional activity in LNCaP and 22Rv1 cells. Data are mean±SEM of *N* = 3 experiments. **G,** Immunofluorescent analysis of AR distribution in LNCaP cells cultured under normoxia or hypoxia (6 hours). Images present a merged image, DAPI staining (Blue), and AR-related fluorescence (Red). **H,** qRT-PCR data demonstrating detectable *KLK3* (PSA) expression in LNCaP, 22Rv1, and CWRR1 cells. Data shown are mean±SEM of *N* = 3 experiments. For all experiments, statistical analysis was carried out using a Student two-tailed *t* test, Mann–Whitney U test, or 2-way ANOVA with Bonferroni post-tests: *, *P <* 0.05; **, *P <* 0.01; ***, *P <* 0.001.

The effects of hypoxia as a mode of resistance is well established for several therapeutic modalities. The impact of environmental hypoxia in modulating the sensitivity to enzalutamide was investigated using a panel of AR-expressing prostate cancer cell lines. Enzalutamide was observed to reduce parental LNCaP viability under normal culture conditions (*P* < 0.05) but was ineffective in hypoxic environments (Fig. 2A). Similarly, the sensitivity of aggressive, LNCaP-derived, C4-2B cells to enzalutamide was markedly reduced under hypoxia (Fig. 2B), with cell-cycle profiling indicating a reduction in Sub-G_0_–G_1_ population and increased detection of cells within the G_2_–M fraction (Fig. 2C).

Further experiments were conducted to determine whether the reduced sensitivity to enzalutamide was associated with the modulation of AR target expression or pathway activation. The effect of hypoxia on AR expression and activity was explored in LNCaP, 22Rv1, and CWR-R1 cell lines. Hypoxia induced a time-dependent increase in the expression of AR protein in all three cell lines over a 24-hour time course, with further evidence of increased AR-V7 expression detected in the 22Rv1 and CWRR-1 lines (Fig. 2D). The effects of hypoxia on AR cellular activity in LNCaP and 22Rv1 cells was investigated by luciferase reporter assays, wherein hypoxia induced a time-dependent increase in ARE-driven luciferase activity in LNCaP cells, but not in 22Rv1 cells (Fig. 2E). The absence of response to hypoxia in 22Rv1 cells was explained by demonstration of high intrinsic levels of AR activity, consistent with the high nuclear concentration detected in these cells (Fig. 2F). Confirmation of AR localization in LNCaP was investigated by immunofluorescence microscopy confirmed that hypoxia exposure increased the nuclear/cytoplasmic distribution of the AR in LNCaP cells (Fig. 2G). Finally, consistent with the sustained AR activation observed in LNCaP cells, we observed a significant elevation in the AR-regulated *KLK3* (PSA) gene expression (Fig. 2H); early onset-changes in *KLK3* mRNA were also detected in hypoxic CWR-R1 cells, while high levels of *KLK3* gene expression were detected consistently at all-time points in 22Rv1 cells (Supplementary Fig. S3A). Our observations are consistent with increased or sustained high AR activity under hypoxic conditions.

### IL8 and VEGF signaling sustain AR pathway activation and modulate therapeutic response in prostate cancer cells and LNCaP tumor xenografts

**Figure 3.**
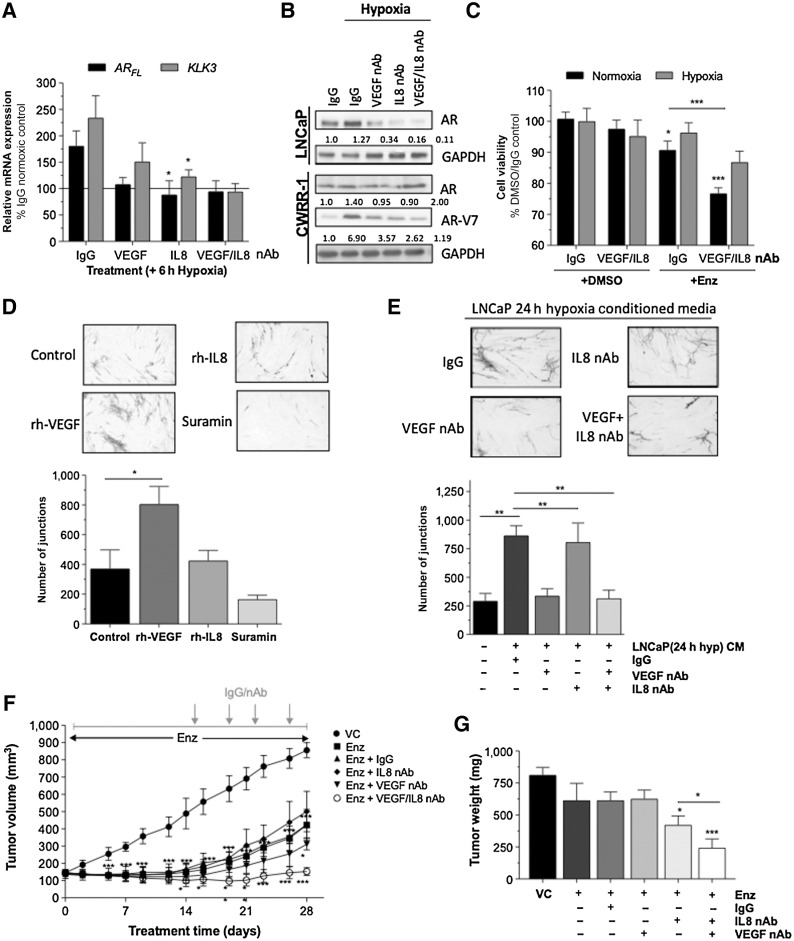
IL8 and VEGF signaling sustain AR pathway activation and modulate response to enzalutamide. Cells were treated with anti-IL8 nAb (5 μg/mL), anti-VEGF nAb (10 μg/mL) or the highest concentration of isotype-matched human IgG antibody. **A,** Effect of anti-IL8 nAb/anti-VEGF nAb on the response of hypoxic or normoxic LNCaP cells to 10 μmol/L Enazlutamide (Enz) over 72 hours. Data shown are mean ± SEM of *N* = 3 experiments. **B,** Effect of anti-IL8 nAb and/or anti-VEGF nAb on hypoxia (6 hours)-induced AR and AR-V7 expression in LNCaP and CWRR1 cells. Blots are representative of *N* = 3 experiments. Equal loading was assessed using GAPDH. Relative expression was determined by densitometry using Image J software. **C,** Effect of 10 μmol/L E (Enz) with anti-IL8 nAb (5 μg/mL), anti-VEGF nAb (10 μg/mL) or the highest concentration of isotype-matched human IgG antibody on viability of LNCaP cells under normoxia and hypoxia for 72 hours. **D,** Effect of VEGF (2 ng/mL) or rhIL8 (3 nmol/L) on tubule formation over 10 days. Suramin (20 μmol/L) was included as a negative control. **E,** Effect of CM harvested from LNCaP cells cultured in hypoxia for 24 hours, in the presence or absence of anti-IL8 nAb and/or anti-VEGF nAb, on tubule formation over 10 days. For both experiments (**D** and **E**), number of junctions was measured using AngioSys 2.0 software. Data are mean±SEM of *N* = 8 fields of view. **F,** Tumor growth data (*N* = 5/group), obtained by measuring tumor volume every 2 days for 28 days. Treatment groups were: vehicle-only (VC); Enz (4 mg/kg); Enz (4 mg/kg) + IgG (150 μg/mL); Enz (4 mg/kg) + anti-VEGF nAb (100 μg/mL); and Enz (4 mg/mL) + anti-VEGF (100 μg/mL) and anti-IL8 (50 μg/mL) nAbs. Treatment schematic is shown above graph. Data points represent mean ± SEM. **G,** Average tumor weights at study completion. Values are mean ± SEM (*N* = 5/group). For all experiments statistical analysis was carried out using Student two-tailed *t* test or Mann–Whitney U test: *, *P* < 0.05; **, *P* < 0.01; ***, *P* < 0.001.

Experiments simulating treatment-induced environmental hypoxia were conducted to characterize the intrinsic and extrinsic effects of VEGF and IL8 signaling. We first sought to characterize the intrinsic effects of these tumor-derived, hypoxia-stimulated cytokines, specifically their contribution to sustained AR pathway activity under hypoxic conditions *in vitro*. Concurrent administration of the anti-VEGF/IL8 nAbs abrogated (Supplementary Fig. S3B and S3C) the acute, hypoxia-induced increases in *AR_FL_* mRNA and *KLK3* (PSA) mRNA (Fig. 3A) and AR_FL_-protein (Fig. 3B; Supplementary Fig. S3D) in LNCaP cells. At the functional level, the coinhibition of IL8 and VEGF signaling also increased the sensitivity of LNCaP cells to enzalutamide under normal and hypoxic culture conditions (*P* < 0.05; Fig. 3C). In contrast, while hypoxia-induced upregulation of AR-V7 was reversed by inhibition of VEGF and IL8 signaling in hypoxic CWR-R1 cells, we observed a concurrent increase in AR_FL_ protein expression (Fig. 3B). Inhibiting IL8 and VEGF signaling had no effects in potentiating the sensitivity of CWR-R1 cells to this enzalutamide (Supplementary Fig. S3E).

Our next experiments sought to characterize the extrinsic effect of tumor-derived IL8 and VEGF signaling on the vascular endothelial cells. Although VEGF and IL8 are considered to be pro-angiogenic *in vivo* (19), only the addition of rhVEGF directly stimulated endothelial tubule formation under conditions employed *in vitro* (*P* < 0.05; Fig. 3D). CM collected from hypoxic LNCaP cells also stimulated tubule formation (*P =* 0.0019; Fig. 3E). However, no angiogenic response was observed by addition of CM harvested from hypoxic LNCaP cells exposed to VEGF nAb (*P* < 0.01) or combined IL8/VEGF nAb's (*P* < 0.01); image analysis confirmed a significant reduction in the number of vascular junctions observed under these conditions. The direct addition of these nAbs to the endothelial cells did not affect tubule formation (Supplementary Fig. S4A), suggesting that the hypoxic tumor cells are the predominant source of these pro-angiogenic factors. Furthermore, we confirmed that the addition of each neutralizing Ab abrogated the detectable levels of the targeted cytokine in the CM of hypoxic LNCaP cells (Supplementary Fig. S4B), however, we also observed that the anti-IL8 nAb partially reversed VEGF secretion, and vice versa, demonstrating the potential interaction between these cytokines.

Finally, we focused on examining the combined impacts of inhibiting the intrinsic and extrinsic effects of IL8 and VEGF signaling on LNCaP tumor growth *in vivo*. Enzalutamide treatment caused 99% tumor control compared with vehicle (*P* < 0.001; Fig. 3F) by day 14 (nadir in oxygen levels), beyond which tumor growth was reestablished. At day 28, enzalutamide had effected a 60% reduction in tumor volume relative to vehicle control–treated tumors (Supplementary Table S2; Fig. 3F). Neutralization of VEGF in combination with enzalutamide from day 14 significantly reduced tumor volume by 76.3% (*P =* 0.0002), while IL8 neutralization alone did not attenuate the response the enzalutamide. However, dual neutralization of IL8 and VEGF from day 14 reduced tumor volume by 98.8% (*P <* 0.0001). Average endpoint tumor weights demonstrated significant reduction following enzalutamide/anti-VEGF (*P =* 0.0144; or enzalutamide/anti-VEGF/IL8 (*P =* 0.0009) compared with vehicle (Fig. 3G). Dual neutralization of IL8/VEGF significantly reduced tumor weight compared with enzalutamide/anti-VEGF (*P =* 0.011), highlighting the importance of coinhibition of IL8 and VEGF to enhance tumor reduction.

### Evidence for IL8 and VEGF in underpinning prostate cancer cell resistance to enzalutamide *in vitro* and *in vivo*

**Figure 4.**
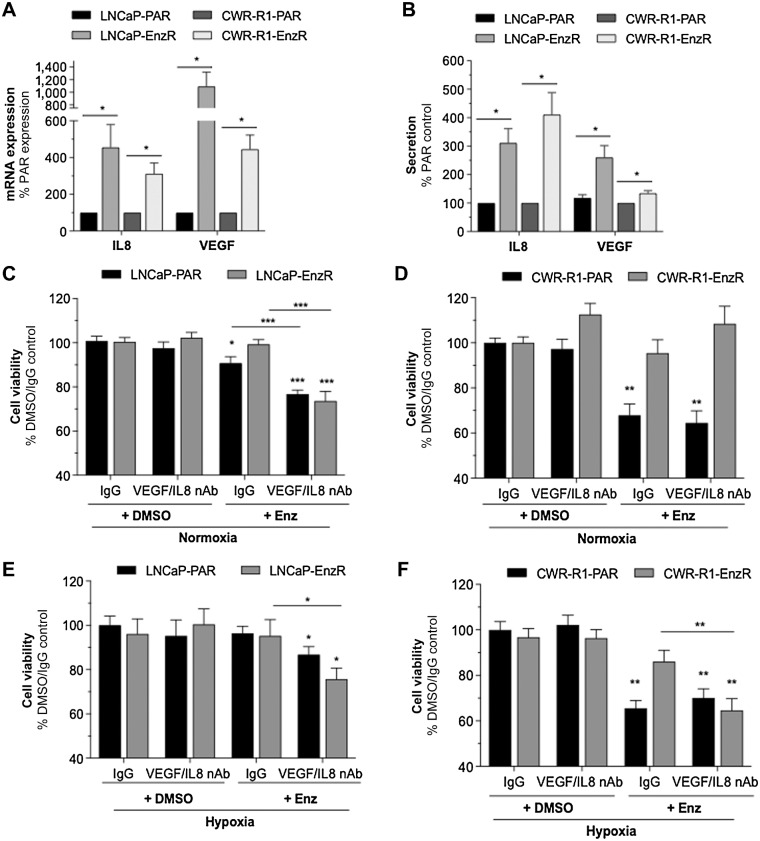
VEGF and IL8 expression is altered and plays a role in resistance in enzalutamide-resistant prostate cancer cell lines. Cells were treated with anti-IL8 nAb (5 μg/mL), anti-VEGF nAb (10 μg/mL), or the highest concentration of isotype-matched human IgG antibody. **A,** qRT-PCR data comparing basal expression of *VEGF*A and *CXCL8* (IL8) expression in LNCaP-Parental (PAR), LNCaP-EnzR, CWR-R1-Par, and CWR-R1-EnzR cell lines. **B,** ELISA data comparing basal secretion of VEGF and IL8 in LNCaP-Par, LNCaP-EnzR, CWR-R1-Par, and CWR-R1-EnzR cells. Data shown are the mean±SEM of *N* = 4 experiments. **C–F,** Bar graphs demonstrating the effect of combined treatment with anti-IL8 nAb and anti-VEGF nAb on the response of LNCaP-Par, LNCaP-EnzR, CWR-R1-Par, and CWR-R1-EnzR cells to 10 μmol/L Enz over 72 hours in (**C** and **D**) normoxia and (**E** and **F**) hypoxia. All experiments data represented as the mean ± SEM of *N* = 3 experiments, unless otherwise stated and statistical analysis was carried out using a Mann-Whitney U test: *, *P <* 0.05; **, *P <* 0.01.

The importance of IL8 and VEGF in underpinning enzalutamide resistance was investigated using isogenic LNCaP and CWR-R1 enzalutamide-sensitive (PAR) and -resistant (EnzR) models (10). AR_FL_ and AR-V7 expression and resistance to enzalutamide was validated (Supplementary Fig. S5A–S5C). Consistent with our hypothesis, VEGF and IL8 mRNA expression (Fig. 4A) and protein secretion (Fig. 4B) were increased in both EnzR models. This was validated in an additional isogenic VCaP EnzR cell line (Supplementary Fig. S5D), and two *in vivo*–generated EnzR models, MR49C and MR49F (20; Supplementary Fig. S5E).

The functional role of elevated IL8 and VEGF signaling as an intrinsic mechanism of resistance was investigated *in vitro*; enzalutamide or neutralization of either IL8 or VEGF alone did not affect the viability of LNCaP-EnzR cells relative to control, but dual neutralization of IL8/VEGF in combination with enzalutamide significantly decreased cell viability by 28% ± 6% (*P* < 0.01%; Supplementary Fig. S6). All further experiments explored the impact of combined VEGF/IL8 neutralization. Under normoxic conditions, dual neutralization of IL8/VEGF potentiated enzalutamide response in both LNCaP-Par and LNCaP-EnzR cells (Fig. 4C) but failed to modulate enzalutamide-sensitivity in normoxic CWR-R1-Par and CWR-R1-EnzR cells (Fig. 4D). However, under hypoxic conditions, neutralization of IL8/VEGF increased sensitivity of Par and EnzR lineages of LNCaP and CWR-R1 cells to enzalutamide, by a factor of 25%±10% in LNCaP-EnzR (*P* < 0.05) and 45% ± 13% in CWR-R1-EnzR cells (*P* < 0.01) relative to corresponding enzalutamide/IgG controls (Fig. 4E and F).

**Figure 5.**
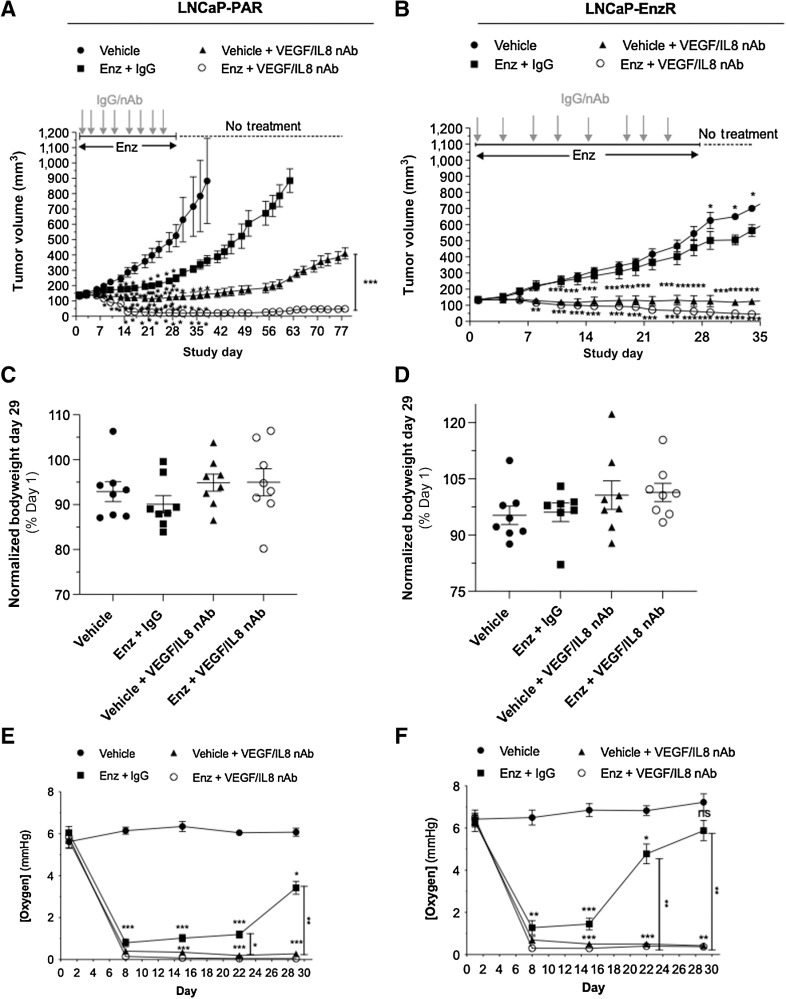
Inhibition of VEGF and IL8 enhances tumor sensitivity to enzalutamide *in vivo.* For all experiments, male *Balb*/c SCID mice bearing tumors of 100 to 150 mm^3^ were assigned to the following treatment groups for 28 days: Vehicle; enzalutamide (Enz; 4 mg/kg) + IgG control (150 μg/mL); Vehicle + anti-VEGF nAb (100 μg/mL) and anti-IL8 (50 μg/mL) nAbs; and Enz (4 mg/mL) + anti-VEGF (100 μg/mL), and anti-IL8 (50 μg/mL) nAbs. **A** and **B,** Tumor growth data, obtained by measuring (**A**) LNCaP-PAR and (**B**) LNCaP-EnzR tumor volume every 2 days. The treatment schematic is shown above the graph. The data points represent the mean ± SD (*N* = 8/group). **C** and **D,** Normalized bodyweight (at end of treatment) of mice with (**C**) LNCaP-PAR and (**D**) LNCaP-EnzR tumors treated with Enz alone or in combination with anti-VEGF nAb and anti-IL8 nAb. Values shown are mean ± SD (*N* = 8/group). **E** and **F,** Intratumoral oxygenation concentration (mmHg) in (**E**) LNCaP-Par and **(F**) LNCaP-EnzR *in vivo* tumors (*N* = 4/group) treated with Enz alone or in combination with anti-VEGF nAb and anti-IL8 nAb for 29 days and measured time-dependent changes. For all experiments, statistical analysis was carried out using a 2-way ANOVA with Tukey post-hoc test: *, *P <* 0.05; **, *P <* 0.01; ***, *P <* 0.001.

Our prior *in vivo* experiments modeled the effects of delayed administration of the anti-VEGF and anti-IL8 antibodies at the time of maximal hypoxia, i.e., day 14 post-enzalutamide treatment. Further experiments were conducted using LNCaP-Par and LNCaP-EnzR xenografts to characterize the impact of concurrent administration of these antibodies with the initiation of enzalutamide treatment over 28 days. As expected, enzalutamide resulted in a significant delay in the rate of LNCaP-Par tumor growth (*P* = 0.0001; Fig. 5A). Dual neutralization of IL8/VEGF resulted in tumor stasis over 28 days, with no detectable growth over the initial tumor volume (Supplementary Table S3; *P* < 0.0001). In contrast, the coadministration of anti-VEGF/anti-IL8 nAbs concurrent with the enzalutamide treatment resulted in the regression of tumors, with 25% being unmeasurable after 20 days, and with no significant change in toxicity compared with enzalutamide alone (Fig. 5A and C). A subgroup of mice was ethically sustained post-therapy to determine whether we could observe evidence of tumor outgrowth or recurrence. While the tumors originally treated with combined IL8/VEGF nAb eventually exhibited outgrowth, tumors subjected to the combined IL8/VEGF nAb and enzalutamide treatment remained static until the study was terminated on day 78 (Fig. 5A).

We further characterized the impact of dual inhibition of IL8/VEGF in the paired LNCaP-EnzR model. As expected, enzalutamide failed to reduce tumor volume compared with controls (Fig. 5B). Consistent with the LNCaP-Par model, there was no significant change in toxicity compared with enzalutamide alone (Fig. 5D). However, of major significance, dual neutralization of IL8/VEGF resulted in tumor stasis from treatment initiation. Interestingly, this was not further enhanced by enzalutamide administration (ANOVA reveals no significant difference between the two treatment cohorts, *P* = 0.6266 with Tukey *post hoc* test), highlighting a novel opportunity for efficacious therapy in an EnzR model.

The effect of enzalutamide on tumor oxygenation was also analyzed for both LNCaP-Par and LNCaP-EnzR xenograft models (Fig. 5E and F). Enzalutamide administration induced a rapid drop in tumor oxygenation reaching a nadir at 8 days in both LNCaP and LNCaP-EnzR models, after which oxygen levels were shown to increase towards pretreatment levels by day 29 (Fig. 5E). The restoration of oxygen levels towards those of vehicle control-treated tumors was observed to be more rapid in the LNCaP-EnzR model than the LNCaP-Par model. However, treatment with either IL8/VEGF nAb alone or in combination with enzalutamide resulted in a sustained drop in tumor oxygenation through to the end of treatment (day 29) in both models.

## Discussion

Following prolonged use of ASIs, many prostate cancers acquire resistance to this mode of therapeutic intervention, and progress to a castrate-resistant state, which is currently incurable, and represents a major clinical unmet need. The current study adds to an increasingly significant body of literature identifying that resistance to AR-targeted therapy may arise, in part, because of inhibiting AR function in the vascular endothelium, as well as within malignant prostate cancer cells. Our study confirms a direct and equivalent cell-cycle inhibitory and cytotoxic effect of enzalutamide on AR-expressing vascular endothelial cells as reported on prostate cancer cells, which is reinforced by its observed capacity to inhibit vascular tubule formation *in vitro*. These effects on vascular endothelial cells are consistent with our *in vivo* observations of a rapid onset and sustained tumor hypoxia following enzalutamide administration to prostate tumor xenografts. The loss of tumor control afforded by enzalutamide, observed at approximately 14 days of therapy, was concurrent with an increased tumor MVD and a rise in oxygen tension, indicative of an environment-promoted angiogenic response. Our results with enzalutamide are consistent with prior imaging of vascular collapse in Bicalutamide-treated tumors, and similar loss of tumor oxygen tension observed in response to finasteride, a 5-alpha-reducatase inhibitor, suggesting that treatment-observed hypoxia is not a drug-specific effect in tumor models, but rather an effect arising from perturbation of androgen signaling in the endothelium (21, 22).

Our experimental approach considered the impacts of microenvironmental hypoxia in modulating the AR signaling pathway in prostate cancer cells. At a molecular level, hypoxia can potentiate AR gene and protein expression, including modulation of both the full-length and variant AR, in a prostate cancer cell specific manner. Hypoxia was also observed to stimulate nuclear translocation and potentiates AR transcriptional activity *in vitro* in LNCaP cells. At a phenotypic level, our data also shows that the therapeutic sensitivity of prostate cancer cells to enzalutamide is reduced under hypoxia. Our data suggests that hypoxia-induced promotion of AR expression and activation may underpin a target-associated resistance to AR-directed therapy intrinsic to tumor cells.

Our key finding of this study is identifying the importance of two hypoxia-regulated cytokines, IL8 and VEGF-A, as critical biological drivers of hypoxia-promoted resistance to AR-targeted therapy. Consistent with prior gene expression profiling of Bicalutamide-resistant tumors (9), we confirmed elevated expression of IL8 and VEGF-A in two independently generated LNCaP-derived EnzR experimental models. While we propose that expression of these factors is induced as a result of developing tumor hypoxia, it is interesting to note the recent characterization of AR-mediated repression of IL8 gene transcription in prostate cancer cell models, indicating that sustained AR inhibition arising from enzalutamide treatment may also directly contribute to increased IL8 production (23).

To our knowledge, this is the first study to demonstrate the importance of their functional cooperativity in prostate cancer *in vivo*, using anti-VEGF/ IL8 nAbs to neutralize their activity in three LNCaP-derived models. Initially, we showed that administration of these antibodies to tumors where hypoxia was already established by continuous AR-targeted therapy (i.e., at the oxygen nadir reached 14 days post initiation of enzalutamide treatment) resulted in a marked delay of LNCaP tumor reoxygenation, increase in MVD and regrowth. Latterly, we observed that administering the anti-VEGF/IL8 nAbs during the period of hypoxia development (i.e., concurrent with initiating enzalutamide therapy) effected a resolution of drug-sensitive LNCaP tumors to below detectable thresholds within 14 days, with sustained tumor control observed for a period extending greater than 30 days post-cessation of treatment, consistent with a durable response. Furthermore, combined inhibition of these hypoxia-regulated factors had pronounced effects even in enzalutamide-resistant LNCaP-derived tumors, either in the absence or presence of enzalutamide, suggesting a potential clinical utility of an anti-VEGF/IL8 therapeutic strategy in tumors that have progressed on enzalutamide therapy. The very significant effects observed with the antibody cocktail alone in this drug-resistant model suggest a potential addiction of these tumors to these cytokines which is essential to tumor adaptation to the hypoxia-conditioned microenvironment.

Hypoxia modulates IL8 and VEGF expression in multiple cell lineages (8, 11, 24, 25), including malignant prostate cancer cells and vascular endothelial cells, suggesting that these cytokine/growth factors may originate from multiple sources within hypoxic zones. Equally, the mechanism/s underpinning the contribution of IL8 and VEGF to therapeutic resistance is likely to be multifactorial and requires further detailed investigation. Our *in vivo* experiments quantifying MVD clearly demonstrate the importance of promoting angiogenesis within the tumors as a major contributing mechanism, consistent with the pro-angiogenic functions of VEGF and IL8. Furthermore, our *in vitro* data demonstrate the association of IL8 and VEGF signaling with regulation of AR expression in hypoxic prostate cancer cells, consistent with these factors contributing to a target-associated resistance. We speculate that further contributing and overlapping mechanisms are probable, including the ability of cooperative IL8 and VEGF signaling to stimulate key transcriptional programs including that of the AR, NF-κB, and HIF-1, resulting in a broad and sustained intrinsic modulation of pro-tumorigenic, resistance-associated, and proliferation-driving genes in tumor and vascular endothelial cells resident within the hypoxic zone (11).

The contribution of these biological drivers in modulating cell plasticity is also possible. The emergence of the ARv7 variant AR isoforms is an established mechanism of enzalutamide resistance in patients (26). We observed that administration of the anti-VEGF/IL8 nAb combination reduces expression of ARv7 in hypoxic CWR-R1 cell lines, suggesting that this intervention may enable resistant cells to reacquire sensitivity to AR-targeted therapies. Furthermore, CXCR2 expression, one of two principal receptors for IL8, has been proposed as a marker and driver of neuroendocrine differentiation (27). Interestingly, we have previously reported a hypoxia-induced upregulation of IL8 and CXCR2 gene expression in prostate cancer cells that prolongs survival under this environmental stress (11). Therefore, a consequence of AR-targeted therapy-induced hypoxia may be to increase CXCR2 expression in tumor cells, which by extension results in the selection of cells undergoing transition to neuroendocrine prostate cancer, a known driver of castration-resistance. A further mechanism worthy of mention is the demonstrated role of these cytokines in immune modulation, specifically cells of myeloid derivation. For example, CXCR2 signaling promotes the infiltration of myeloid-derived suppressor cells (28) and preferentially sustains pro-tumorigenic M2-differentiation of tumor-resident macrophages within the TME of murine PTEN-deficient prostate cancer's (29).

Clinical investigation of angiogenesis inhibitors has been studied in advanced prostate cancer. Phase III trials of anti-angiogenic compounds including Bevacizumab, Sunitinib, and Lenalidomide were discontinued due to poor efficacy in metastatic CRPC (30). The clinical failure of these highly selective anti-angiogenic agents in heterogeneous diseases such as metastatic CRPC may arise due to their narrow target profile and/or the subsequent inability to target other key factors that play a role in regulating the tumor response to hypoxia. Specifically, the chemokine IL8 has been shown to underpin the angiogenic response of HIF-1α–deficient/VEGF-depleted DLD-1 colon cancer xenografts (31), and mediate the resistance of head and neck squamous cell carcinoma and renal cell carcinoma to Bevacizumab (32) and Sunitinib (33), respectively. IL8 expression is clinically relevant in PC and correlates not only with MVD, but also with shorter time-to-progression for patients being treated with hormone therapy (34, 35). As demonstrated by our preclinical data, targeting VEGF alone afforded only temporary and suboptimal tumor control. Instead, cotargeting of both VEGF and IL8 signaling was essential in achieving the greatest magnitude and duration of tumor control *in vivo*. Therefore, in consideration of past clinical experience and our current observations, a concurrent inhibition of these signaling factors is most likely required to eradicate the biological redundancy and offer maximal and durable clinical responses. Moreover, it is important to note that our use of an antihuman IL8 nAb restricts its action in our *in vivo* experimental models to targeting the effects of IL8 secreted from human LNCaP model. Consequently, we are not accounting for the actions of hypoxia-induced murine CXC-chemokine ligands (CXCL1, CXCL2, and CXCL5) originating from murine stromal and vascular endothelial cells which remains intact and therefore may provide some degree of functional compensation. As such, administration of pharmacologic agents that provide total disruption of hypoxia-induced ELR^+^ chemokine signaling within the prostate TME, and in the presence of a fully competent and humanized immune system may yet provide even more impressive results.

In conclusion, our study defines a further mode of microenvironment-mediated resistance, which arises as a consequence of treatment-induced collapse of the vascular supply to prostate tumors. This mode of resistance is distinct from the numerous modes of intrinsic resistance mechanisms and confirms that the impact of inhibiting androgen signaling extends beyond that of CAFs within the TME. Our demonstration that combined VEGF/IL8 inhibition extends the duration of sensitivity of prostate tumors to enzalutamide *in vivo* and restores sensitivity of drug-resistant models encourages future evaluation in further experimental models, and potential translation of this discovery to proof-of-concept trials. Moreover, our results provide further insights into the complex nature and multi-targeted therapeutic regimens that may be required to prolong the clinical benefit of next-generation AR antagonists in men with advanced, life-threatening prostate cancer.

We show treatment-promoted hypoxia and microenvironment adaptation facilitates resistance to enzalutamide. Concurrent inhibition of two hypoxia-induced factors, IL8 and VEGF-A, prolongs tumor sensitivity to enzalutamide in preclinical models. Targeting hypoxia-induced signaling may extend the therapeutic benefit of enzalutamide and provide an improved treatment strategy for patients with resistant disease.

## Supplementary Material

Supplementary Figure
