# Supplementary material for: Attenuating Adaptive VEGF-A and IL8 Signaling Restores Durable Tumor Control in AR Antagonist–Treated Prostate Cancers
Source: Mol Cancer Res. 2022 Mar 18;20(6):841–53. doi: 10.1158/1541-7786.MCR-21-0780 (PMC9381111; doi:10.1158/1541-7786.MCR-21-0780)
Supplement: Supplementary Figure [file mcr-21-0780_supplementary_tables_1-3_supplementary_figures_1-6_suppsm1-6.pptx]

## Slide 1
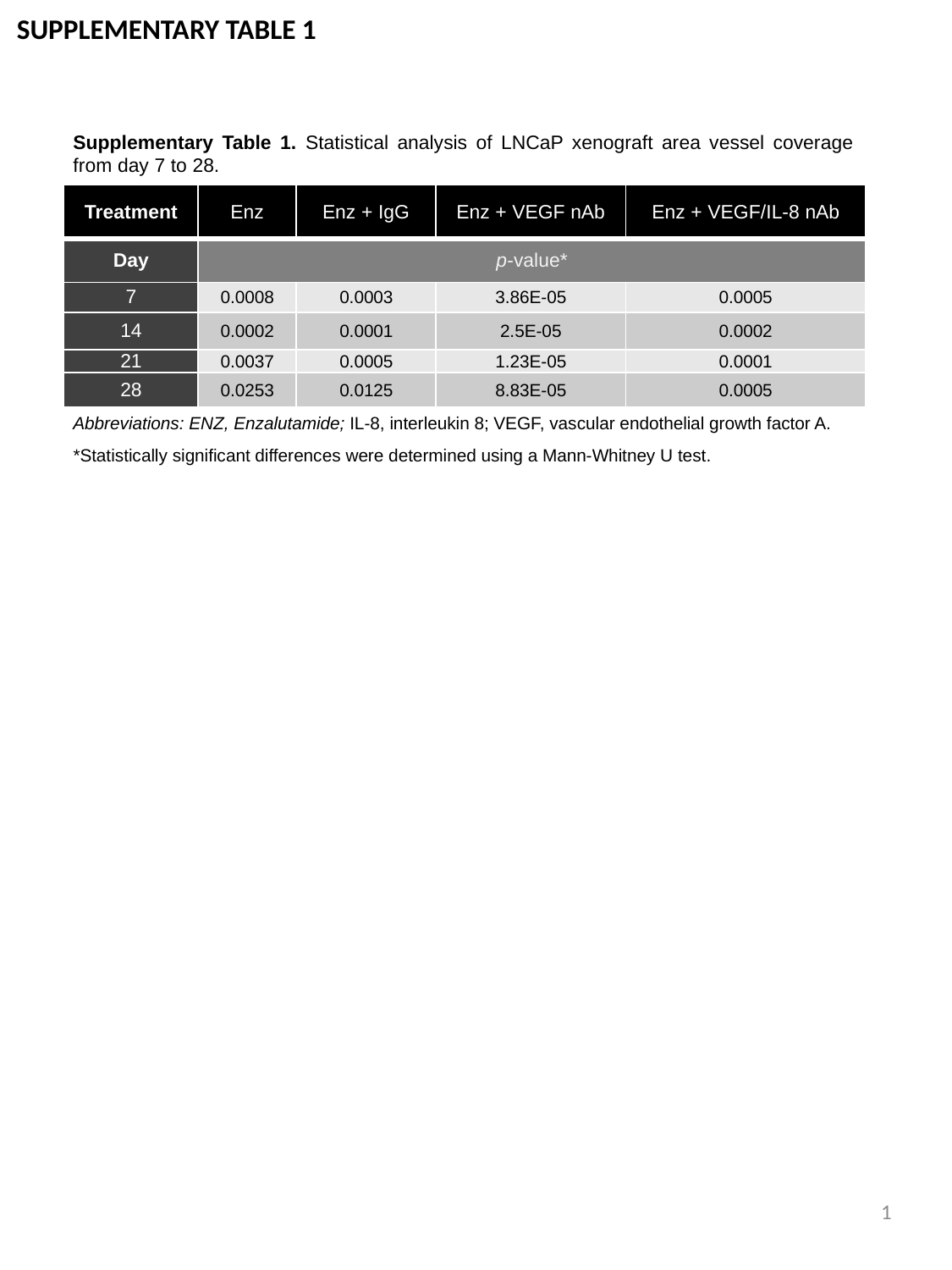

SUPPLEMENTARY TABLE 1
Supplementary Table 1. Statistical analysis of LNCaP xenograft area vessel coverage from day 7 to 28.
| Treatment | Enz | Enz + IgG | Enz + VEGF nAb | Enz + VEGF/IL-8 nAb |
| --- | --- | --- | --- | --- |
| Day | p-value\* | | | |
| 7 | 0.0008 | 0.0003 | 3.86E-05 | 0.0005 |
| 14 | 0.0002 | 0.0001 | 2.5E-05 | 0.0002 |
| 21 | 0.0037 | 0.0005 | 1.23E-05 | 0.0001 |
| 28 | 0.0253 | 0.0125 | 8.83E-05 | 0.0005 |
Abbreviations: ENZ, Enzalutamide; IL-8, interleukin 8; VEGF, vascular endothelial growth factor A.
*Statistically significant differences were determined using a Mann-Whitney U test.
1

## Slide 2
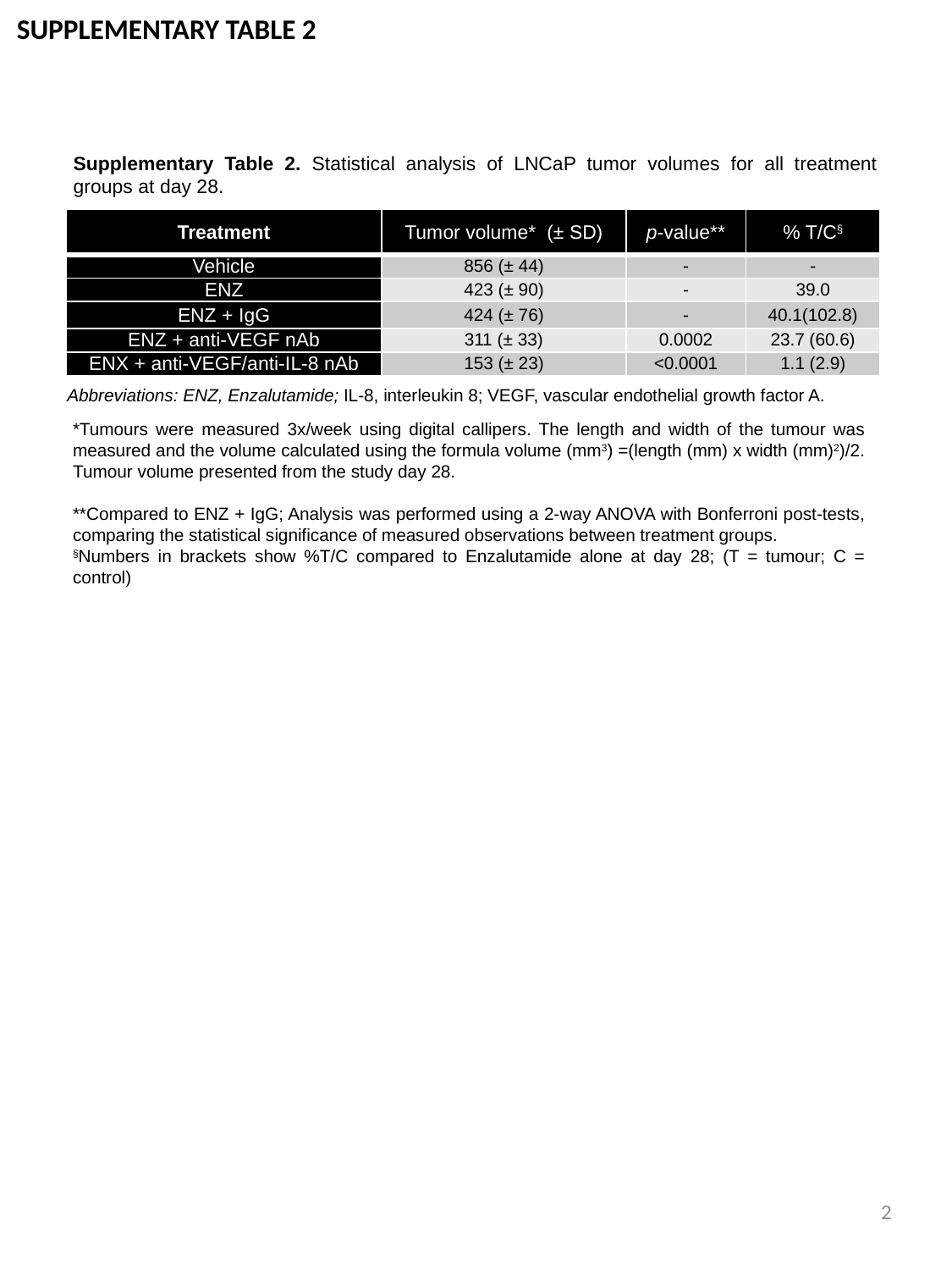

SUPPLEMENTARY TABLE 2
Supplementary Table 2. Statistical analysis of LNCaP tumor volumes for all treatment groups at day 28.
| Treatment | Tumor volume\* (± SD) | p-value\*\* | % T/C§ |
| --- | --- | --- | --- |
| Vehicle | 856 (± 44) | - | - |
| ENZ | 423 (± 90) | - | 39.0 |
| ENZ + IgG | 424 (± 76) | - | 40.1(102.8) |
| ENZ + anti-VEGF nAb | 311 (± 33) | 0.0002 | 23.7 (60.6) |
| ENX + anti-VEGF/anti-IL-8 nAb | 153 (± 23) | <0.0001 | 1.1 (2.9) |
Abbreviations: ENZ, Enzalutamide; IL-8, interleukin 8; VEGF, vascular endothelial growth factor A.
*Tumours were measured 3x/week using digital callipers. The length and width of the tumour was measured and the volume calculated using the formula volume (mm3) =(length (mm) x width (mm)2)/2. Tumour volume presented from the study day 28.
**Compared to ENZ + IgG; Analysis was performed using a 2-way ANOVA with Bonferroni post-tests, comparing the statistical significance of measured observations between treatment groups.
§Numbers in brackets show %T/C compared to Enzalutamide alone at day 28; (T = tumour; C = control)
2

## Slide 3
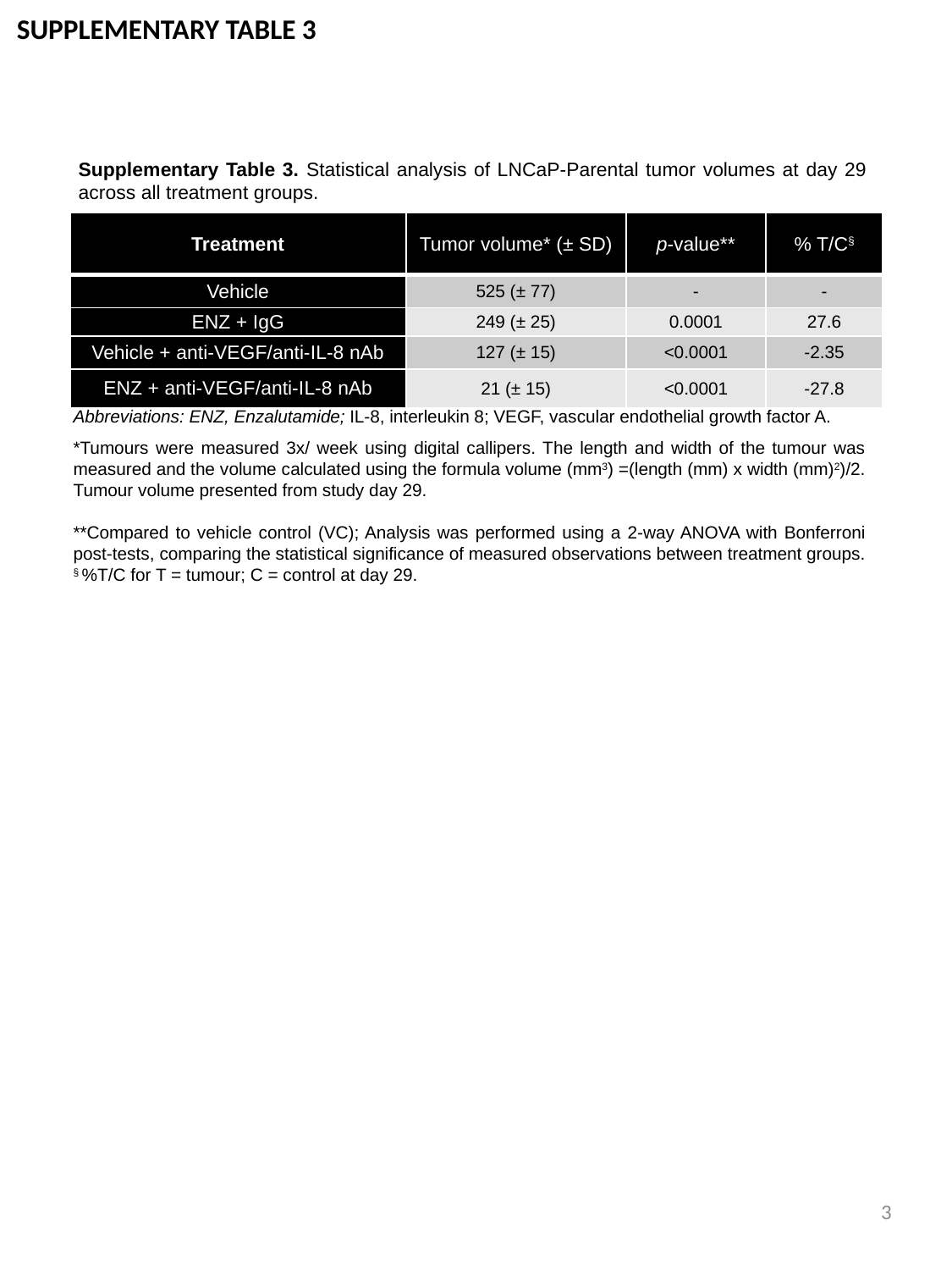

SUPPLEMENTARY TABLE 3
Supplementary Table 3. Statistical analysis of LNCaP-Parental tumor volumes at day 29 across all treatment groups.
| Treatment | Tumor volume\* (± SD) | p-value\*\* | % T/C§ |
| --- | --- | --- | --- |
| Vehicle | 525 (± 77) | - | - |
| ENZ + IgG | 249 (± 25) | 0.0001 | 27.6 |
| Vehicle + anti-VEGF/anti-IL-8 nAb | 127 (± 15) | <0.0001 | -2.35 |
| ENZ + anti-VEGF/anti-IL-8 nAb | 21 (± 15) | <0.0001 | -27.8 |
Abbreviations: ENZ, Enzalutamide; IL-8, interleukin 8; VEGF, vascular endothelial growth factor A.
*Tumours were measured 3x/ week using digital callipers. The length and width of the tumour was measured and the volume calculated using the formula volume (mm3) =(length (mm) x width (mm)2)/2. Tumour volume presented from study day 29.
**Compared to vehicle control (VC); Analysis was performed using a 2-way ANOVA with Bonferroni post-tests, comparing the statistical significance of measured observations between treatment groups.
§ %T/C for T = tumour; C = control at day 29.
3

## Slide 4
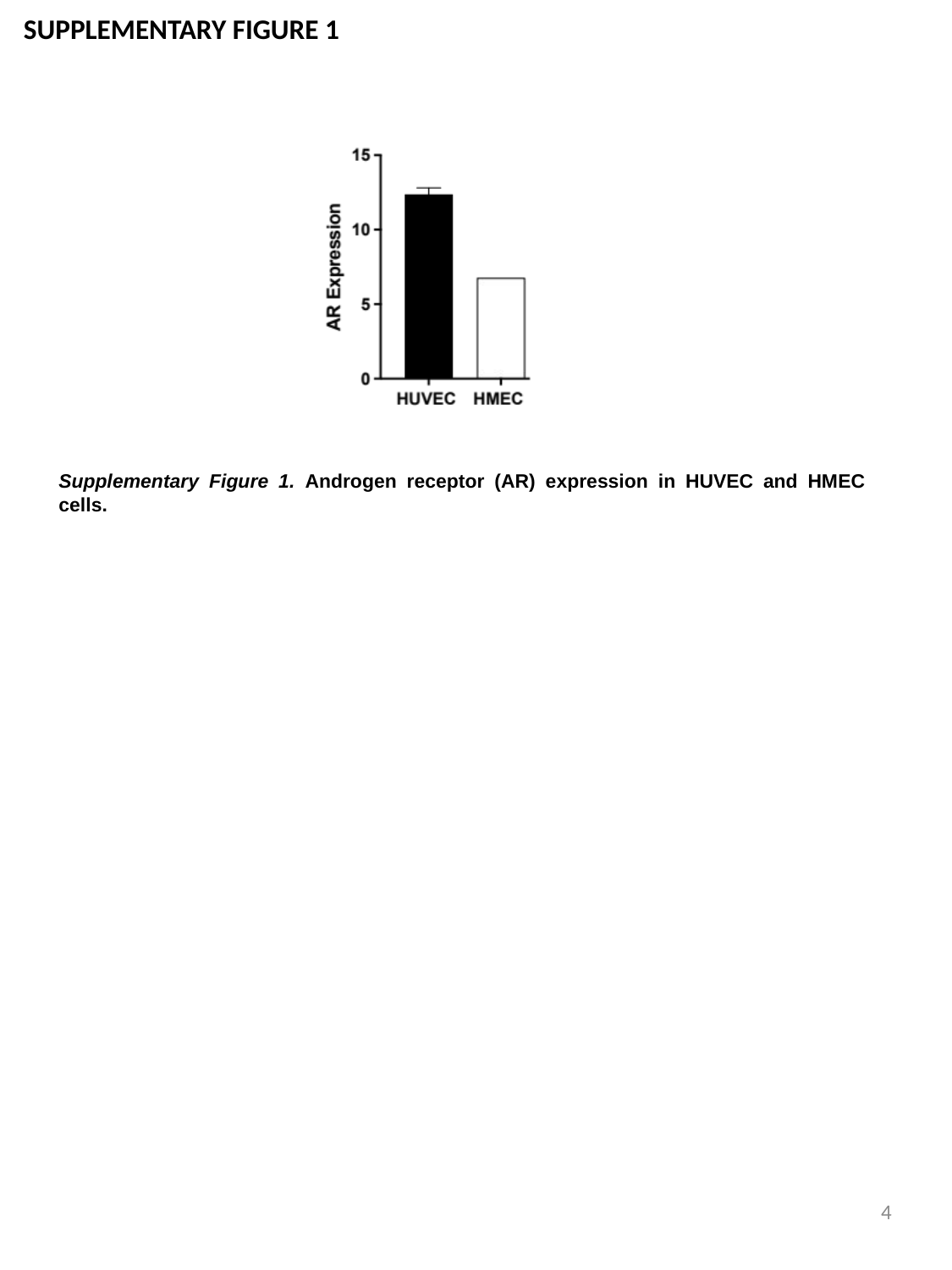

SUPPLEMENTARY FIGURE 1
Supplementary Figure 1. Androgen receptor (AR) expression in HUVEC and HMEC cells.
4

## Slide 5
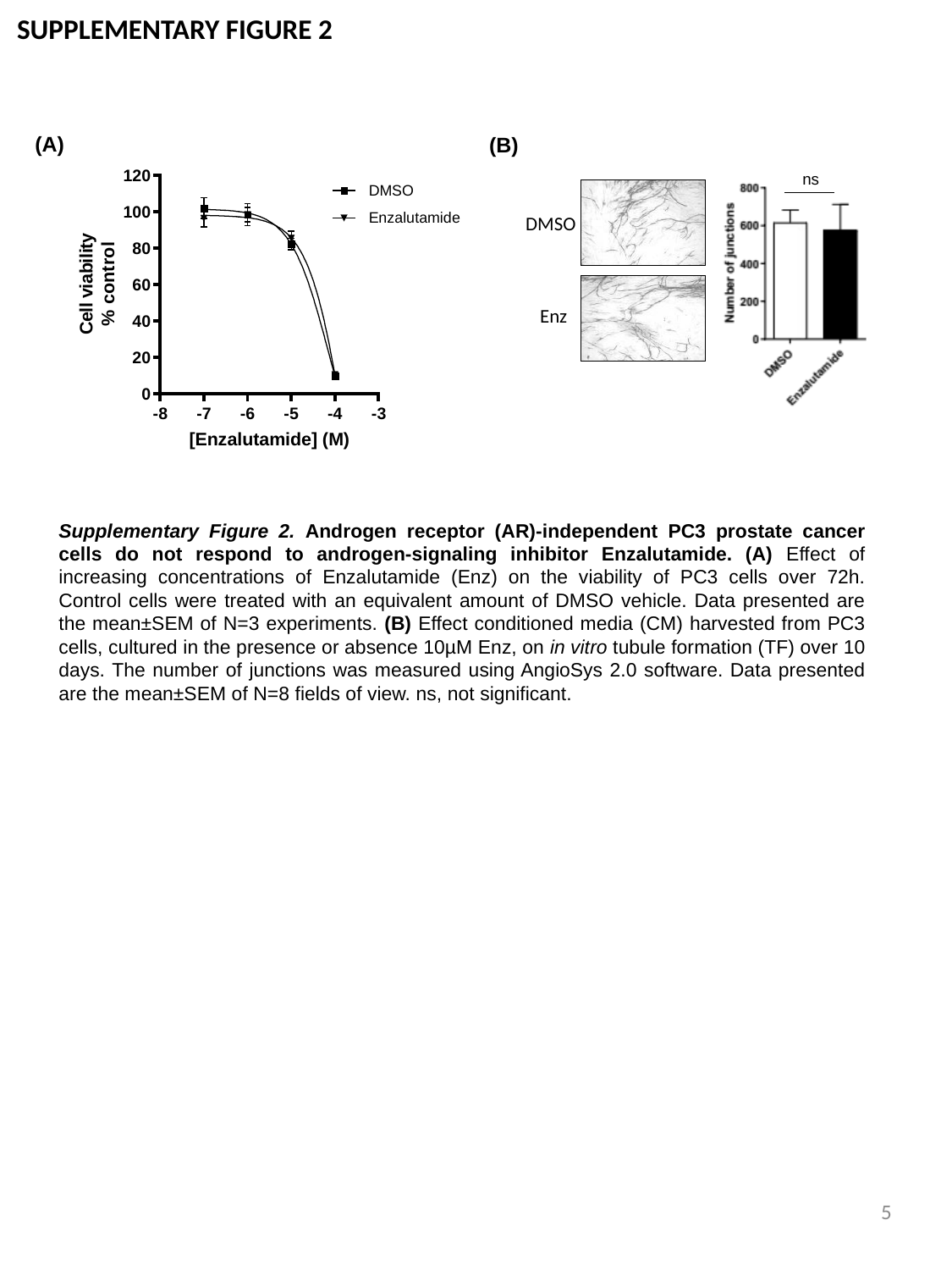

SUPPLEMENTARY FIGURE 2
(A)
(B)
ns
DMSO
Enz
Supplementary Figure 2. Androgen receptor (AR)-independent PC3 prostate cancer cells do not respond to androgen-signaling inhibitor Enzalutamide. (A) Effect of increasing concentrations of Enzalutamide (Enz) on the viability of PC3 cells over 72h. Control cells were treated with an equivalent amount of DMSO vehicle. Data presented are the mean±SEM of N=3 experiments. (B) Effect conditioned media (CM) harvested from PC3 cells, cultured in the presence or absence 10µM Enz, on in vitro tubule formation (TF) over 10 days. The number of junctions was measured using AngioSys 2.0 software. Data presented are the mean±SEM of N=8 fields of view. ns, not significant.
5

## Slide 6
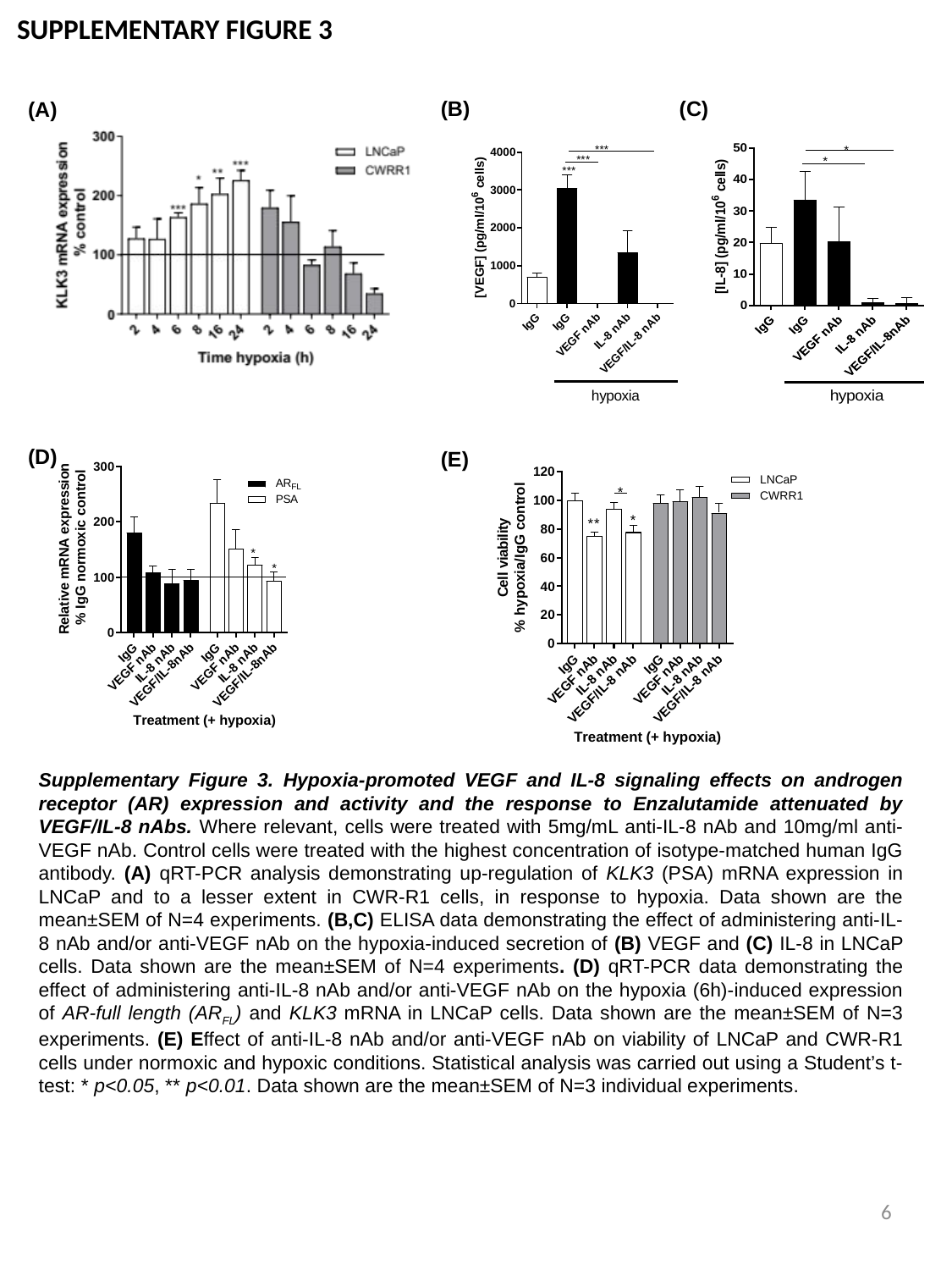

SUPPLEMENTARY FIGURE 3
(B)
(C)
(A)
(D)
(E)
Supplementary Figure 3. Hypoxia-promoted VEGF and IL-8 signaling effects on androgen receptor (AR) expression and activity and the response to Enzalutamide attenuated by VEGF/IL-8 nAbs. Where relevant, cells were treated with 5mg/mL anti-IL-8 nAb and 10mg/ml anti-VEGF nAb. Control cells were treated with the highest concentration of isotype-matched human IgG antibody. (A) qRT-PCR analysis demonstrating up-regulation of KLK3 (PSA) mRNA expression in LNCaP and to a lesser extent in CWR-R1 cells, in response to hypoxia. Data shown are the mean±SEM of N=4 experiments. (B,C) ELISA data demonstrating the effect of administering anti-IL-8 nAb and/or anti-VEGF nAb on the hypoxia-induced secretion of (B) VEGF and (C) IL-8 in LNCaP cells. Data shown are the mean±SEM of N=4 experiments. (D) qRT-PCR data demonstrating the effect of administering anti-IL-8 nAb and/or anti-VEGF nAb on the hypoxia (6h)-induced expression of AR-full length (ARFL) and KLK3 mRNA in LNCaP cells. Data shown are the mean±SEM of N=3 experiments. (E) Effect of anti-IL-8 nAb and/or anti-VEGF nAb on viability of LNCaP and CWR-R1 cells under normoxic and hypoxic conditions. Statistical analysis was carried out using a Student’s t-test: * p<0.05, ** p<0.01. Data shown are the mean±SEM of N=3 individual experiments.
6

## Slide 7
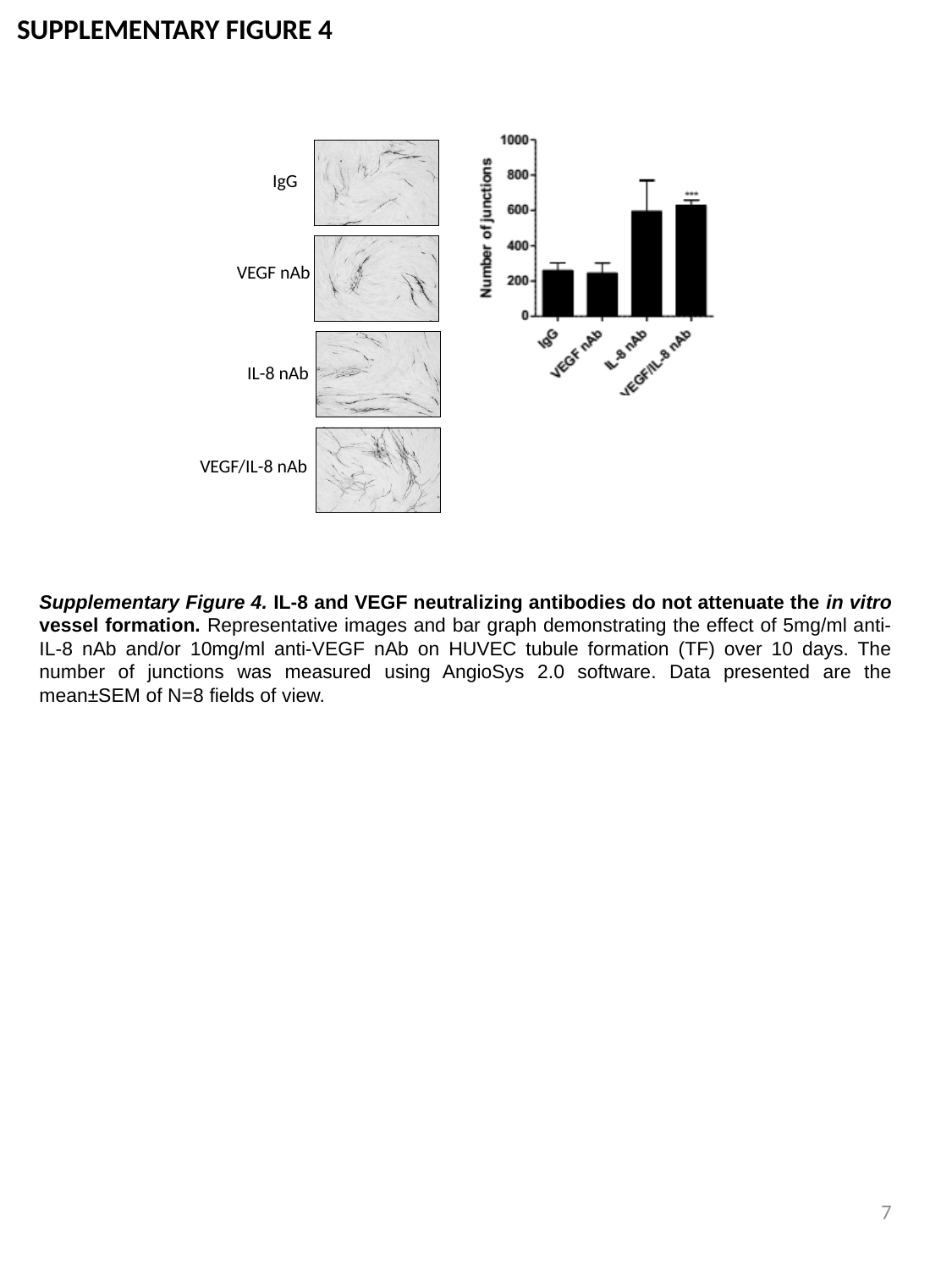

SUPPLEMENTARY FIGURE 4
IgG
VEGF nAb
IL-8 nAb
VEGF/IL-8 nAb
Supplementary Figure 4. IL-8 and VEGF neutralizing antibodies do not attenuate the in vitro vessel formation. Representative images and bar graph demonstrating the effect of 5mg/ml anti-IL-8 nAb and/or 10mg/ml anti-VEGF nAb on HUVEC tubule formation (TF) over 10 days. The number of junctions was measured using AngioSys 2.0 software. Data presented are the mean±SEM of N=8 fields of view.
7

## Slide 8
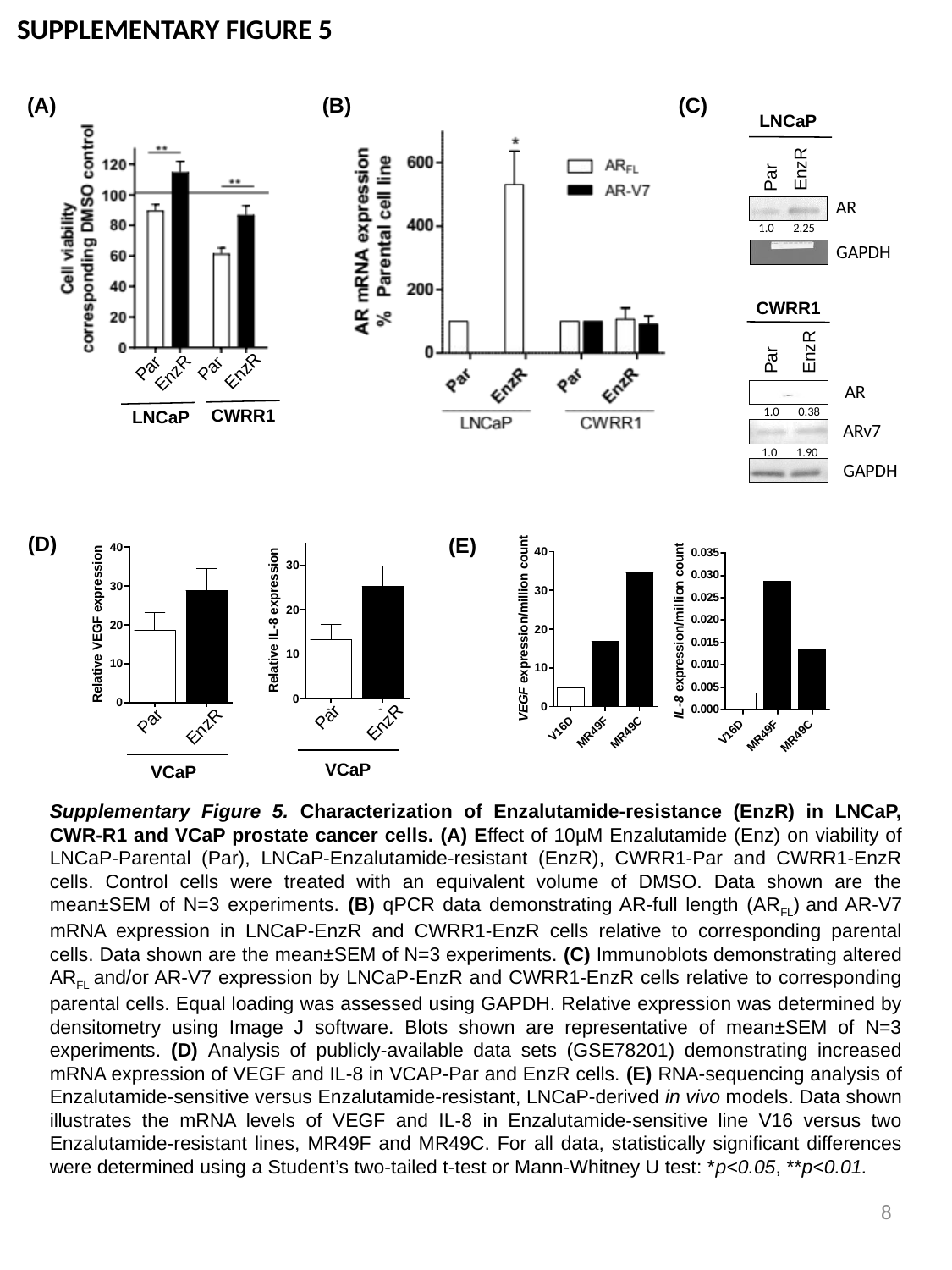

SUPPLEMENTARY FIGURE 5
(C)
(A)
(B)
LNCaP-EnzR
LNCaP-Par
AR
GAPDH
1.0 2.25
LNCaP
EnzR
Par
CWRR1-EnzR
CWRR1-Par
AR
ARv7
GAPDH
1.0 0.38
1.0 1.90
CWRR1
EnzR
Par
Par
Par
EnzR
EnzR
CWRR1
LNCaP
(D)
(E)
Par
Par
EnzR
EnzR
VCaP
VCaP
Supplementary Figure 5. Characterization of Enzalutamide-resistance (EnzR) in LNCaP, CWR-R1 and VCaP prostate cancer cells. (A) Effect of 10µM Enzalutamide (Enz) on viability of LNCaP-Parental (Par), LNCaP-Enzalutamide-resistant (EnzR), CWRR1-Par and CWRR1-EnzR cells. Control cells were treated with an equivalent volume of DMSO. Data shown are the mean±SEM of N=3 experiments. (B) qPCR data demonstrating AR-full length (ARFL) and AR-V7 mRNA expression in LNCaP-EnzR and CWRR1-EnzR cells relative to corresponding parental cells. Data shown are the mean±SEM of N=3 experiments. (C) Immunoblots demonstrating altered ARFL and/or AR-V7 expression by LNCaP-EnzR and CWRR1-EnzR cells relative to corresponding parental cells. Equal loading was assessed using GAPDH. Relative expression was determined by densitometry using Image J software. Blots shown are representative of mean±SEM of N=3 experiments. (D) Analysis of publicly-available data sets (GSE78201) demonstrating increased mRNA expression of VEGF and IL-8 in VCAP-Par and EnzR cells. (E) RNA-sequencing analysis of Enzalutamide-sensitive versus Enzalutamide-resistant, LNCaP-derived in vivo models. Data shown illustrates the mRNA levels of VEGF and IL-8 in Enzalutamide-sensitive line V16 versus two Enzalutamide-resistant lines, MR49F and MR49C. For all data, statistically significant differences were determined using a Student’s two-tailed t-test or Mann-Whitney U test: *p<0.05, **p<0.01.
8

## Slide 9
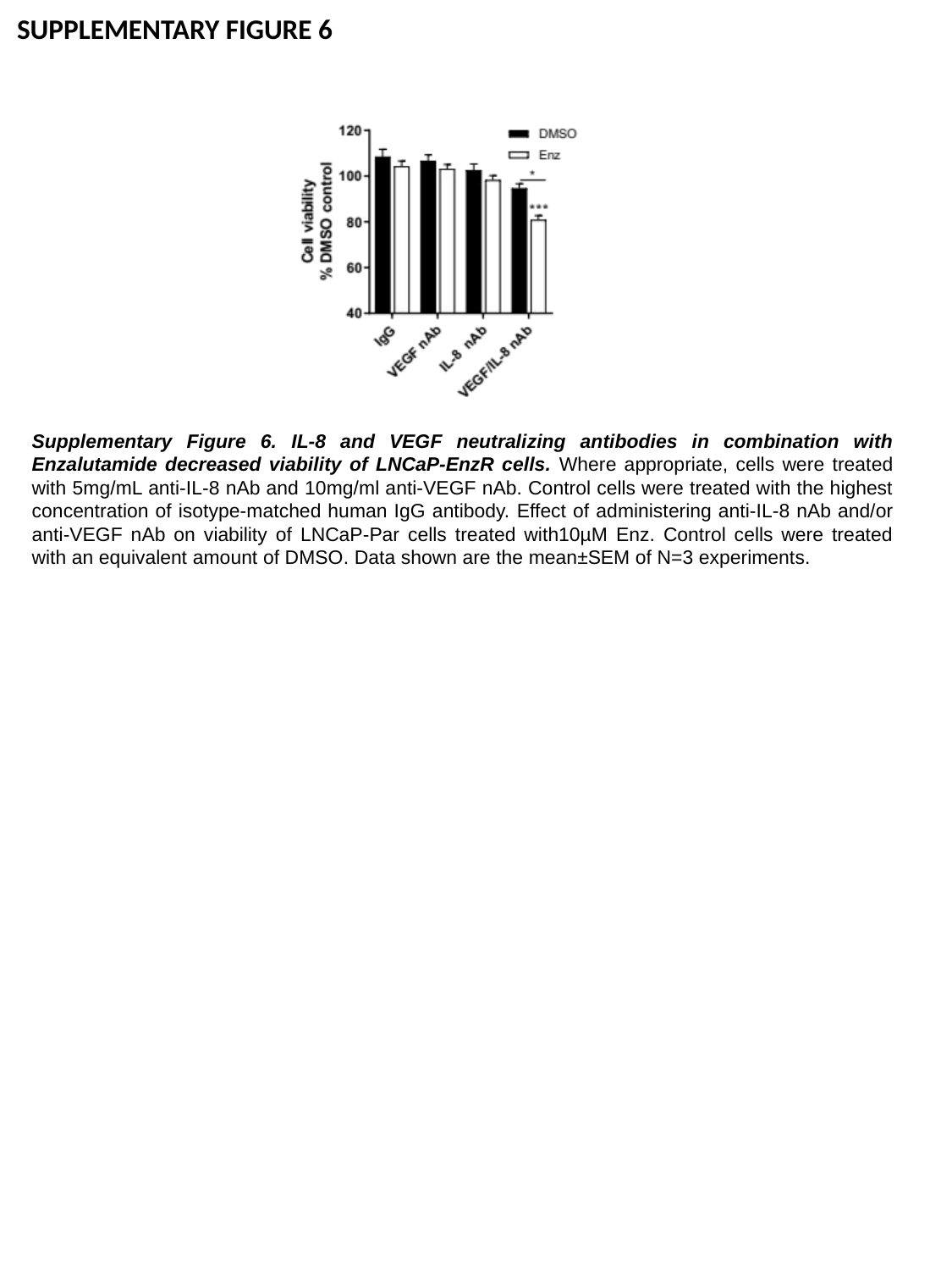

SUPPLEMENTARY FIGURE 6
Supplementary Figure 6. IL-8 and VEGF neutralizing antibodies in combination with Enzalutamide decreased viability of LNCaP-EnzR cells. Where appropriate, cells were treated with 5mg/mL anti-IL-8 nAb and 10mg/ml anti-VEGF nAb. Control cells were treated with the highest concentration of isotype-matched human IgG antibody. Effect of administering anti-IL-8 nAb and/or anti-VEGF nAb on viability of LNCaP-Par cells treated with10µM Enz. Control cells were treated with an equivalent amount of DMSO. Data shown are the mean±SEM of N=3 experiments.

## Slide 10
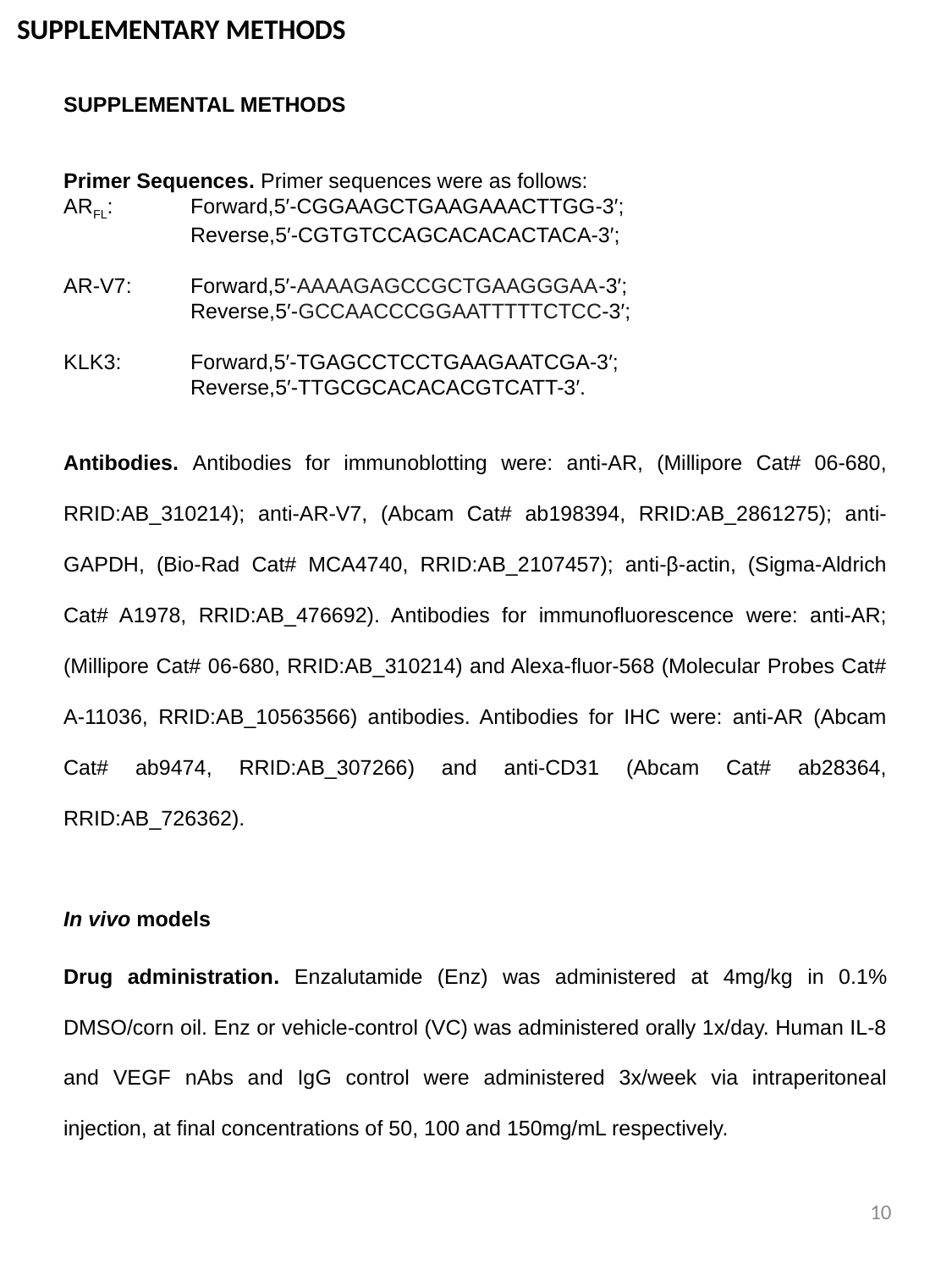

SUPPLEMENTARY METHODS
SUPPLEMENTAL METHODS
Primer Sequences. Primer sequences were as follows:
ARFL: 	Forward,5′-CGGAAGCTGAAGAAACTTGG-3′;
	Reverse,5′-CGTGTCCAGCACACACTACA-3′;
AR-V7: 	Forward,5′-AAAAGAGCCGCTGAAGGGAA-3′;
	Reverse,5′-GCCAACCCGGAATTTTTCTCC-3′;
KLK3: 	Forward,5′-TGAGCCTCCTGAAGAATCGA-3′;
	Reverse,5′-TTGCGCACACACGTCATT-3′.
Antibodies. Antibodies for immunoblotting were: anti-AR, (Millipore Cat# 06-680, RRID:AB_310214); anti-AR-V7, (Abcam Cat# ab198394, RRID:AB_2861275); anti-GAPDH, (Bio-Rad Cat# MCA4740, RRID:AB_2107457); anti-β-actin, (Sigma-Aldrich Cat# A1978, RRID:AB_476692). Antibodies for immunofluorescence were: anti-AR; (Millipore Cat# 06-680, RRID:AB_310214) and Alexa-fluor-568 (Molecular Probes Cat# A-11036, RRID:AB_10563566) antibodies. Antibodies for IHC were: anti-AR (Abcam Cat# ab9474, RRID:AB_307266) and anti-CD31 (Abcam Cat# ab28364, RRID:AB_726362).
In vivo models
Drug administration. Enzalutamide (Enz) was administered at 4mg/kg in 0.1% DMSO/corn oil. Enz or vehicle-control (VC) was administered orally 1x/day. Human IL-8 and VEGF nAbs and IgG control were administered 3x/week via intraperitoneal injection, at final concentrations of 50, 100 and 150mg/mL respectively.
10

## Slide 11
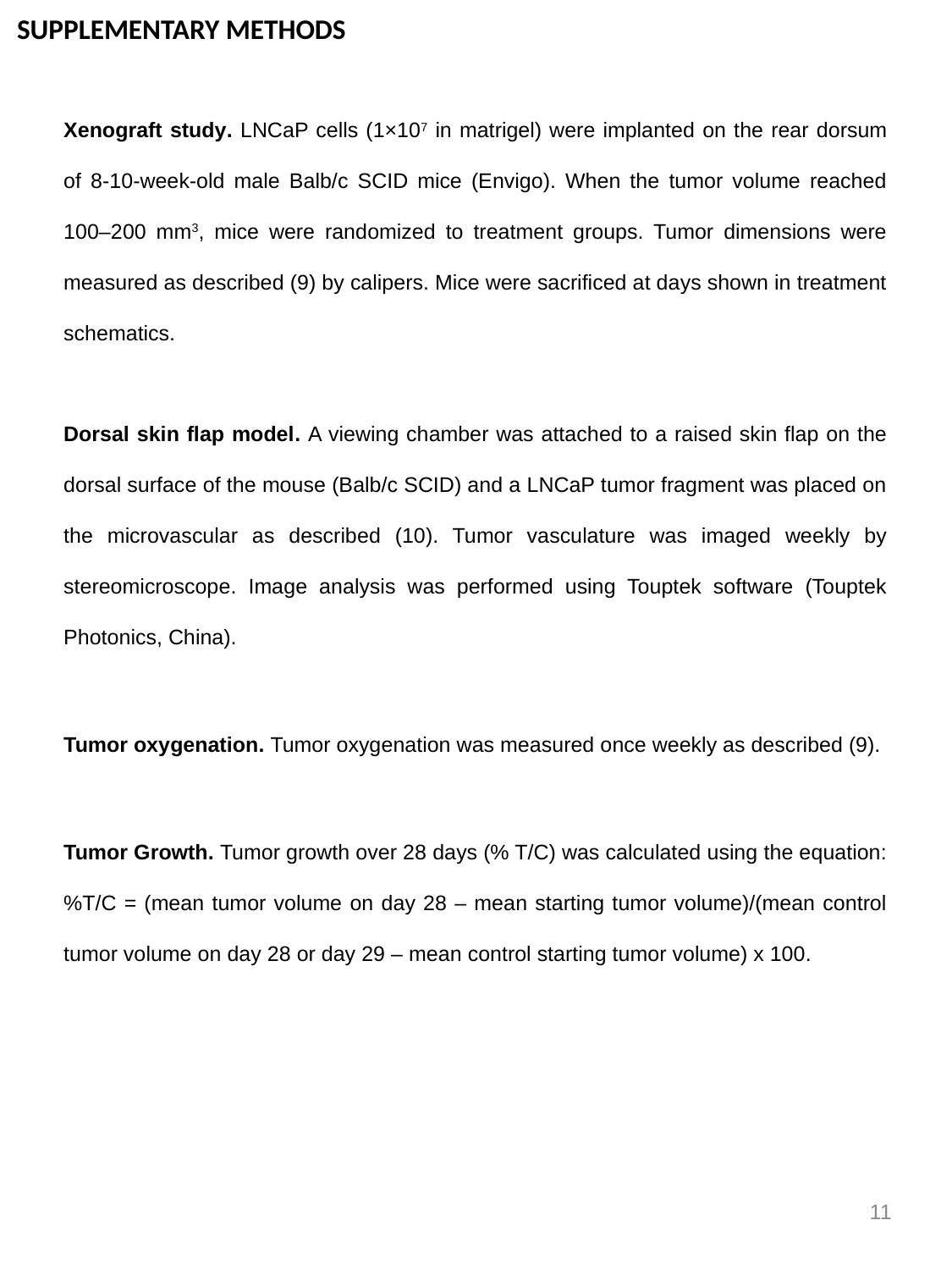

SUPPLEMENTARY METHODS
Xenograft study. LNCaP cells (1×10^7^ in matrigel) were implanted on the rear dorsum of 8-10-week-old male Balb/c SCID mice (Envigo). When the tumor volume reached 100–200 mm3, mice were randomized to treatment groups. Tumor dimensions were measured as described (9) by calipers. Mice were sacrificed at days shown in treatment schematics.
Dorsal skin flap model. A viewing chamber was attached to a raised skin flap on the dorsal surface of the mouse (Balb/c SCID) and a LNCaP tumor fragment was placed on the microvascular as described (10). Tumor vasculature was imaged weekly by stereomicroscope. Image analysis was performed using Touptek software (Touptek Photonics, China).
Tumor oxygenation. Tumor oxygenation was measured once weekly as described (9).
Tumor Growth. Tumor growth over 28 days (% T/C) was calculated using the equation: %T/C = (mean tumor volume on day 28 – mean starting tumor volume)/(mean control tumor volume on day 28 or day 29 – mean control starting tumor volume) x 100.
11
